# Decoding Spatial Heterogeneity and Multi‐Omics Regulation with Hierarchical Graph Learning

**DOI:** 10.1002/advs.75574

**Published:** 2026-05-10

**Authors:** Jiazhou Chen, Jiahui Xie, Yi Liao, Junyu Li, Longqi Liu, Xin Gao, Hongmin Cai

**Affiliations:** ^1^ School of Computer Science and Technology Guangdong University of Technology Guangdong China; ^2^ School of Computer Science and Technology South China University of Technology Guangdong China; ^3^ School of Future Technology South China University of Technology Guangdong China; ^4^ BGI‐Shenzhen Shenzhen China; ^5^ Computer Science Program, Computer, Electrical and Mathematical Sciences and Engineering Division King Abdullah University of Science and Technology (KAUST) King Abdullah Kingdom of Saudi Arabia; ^6^ Center of Excellence for Smart Health (KCSH) King Abdullah University of Science and Technology King Abdullah Kingdom of Saudi Arabia; ^7^ Center of Excellence on Generative AI King Abdullah University of Science and Technology King Abdullah Kingdom of Saudi Arabia

**Keywords:** Graph contrastive learning, Graph representation learning, Molecular regulatory network, Spatial domain identification

## Abstract

Recent advances in spatial multi‐omics technologies have enabled the simultaneous profiling of multiple molecular layers within the same tissue slice, providing unprecedented opportunities to investigate tissue spatial organization. However, most existing computational methods identify spatial domains in a purely data‐driven manner, rarely uncovering the interpretable multi‐omics regulatory mechanisms that underlie spatial heterogeneity. Here, we introduce SMOReg, a spatial multi‐omics hierarchical graph learning framework that embeds cross‐omics regulatory patterns into spot representations via cross‐graph matching, thereby yielding fine‐grained spatial domains with enhanced biological interpretability. Evaluated on paired spatial transcriptomic and proteomic datasets from diverse tissues, SMOReg consistently outperformed existing methods across multiple metrics. Notably, SMOReg uncovered fine‐grained spatial domains characterized by biologically interpretable multi‐layer regulatory signatures that competing approaches overlooked. It successfully distinguished the germinal center light zone and dark zone in human tonsil, which are histologically well‐established yet spatially ambiguous compartments, and revealed their specialized regulatory pathways driving B‐cell proliferation and affinity maturation. SMOReg thus provides a powerful framework for deciphering the interplay between spatial domain heterogeneity and underlying multi‐omics regulation, significantly advancing the interpretability of spatial domains in complex tissues.

## Abstract 2

SMOReg introduces a hierarchical graph learning framework to decode spatial heterogeneity and molecular regulation from spatial multi‐omics data. By integrating biological priors with dual spatial‐feature contexts, the model accurately delineates complex spatial domains. Furthermore, a novel cross‐graph matching mechanism dynamically infers domain‐specific gene‐protein interactions, offering deep, interpretable insights into tissue microenvironments.

## 1 Introduction

The spatial arrangement of cells is closely related to the biological functions of tissues. Cells are not randomly distributed within tissues. Instead, they tend to form various spatial domains, each with its own distinct cellular morphology, molecular composition, and signaling pathways. Typically wrapped in structural hierarchies and boundaries, these domains serve as the fundamental functional units that support diverse physiological activities at the tissue level [1]. Crucially, they also provide a specialized environment for local cellular communication. This facilitates niche‐specific immune regulation and spatially restricted lineage commitment, both of which cannot be fully observed by analyzing cell type composition alone [2]. Consequently, it is the decoding of these domains that lies at the heart of comprehending tissue physiology. Such significance becomes even more pronounced in pathological contexts, where the structural remodeling or breakdown of these spatial compartments frequently propels conditions such as tumor growth and persistent inflammation [3].

Spatial transcriptomics has become a subject of significant research concern due to its capability to enable genome‐wide measurement of gene expression in situ. By employing spatial transcriptomics, the intricate interplay between cellular heterogeneity and tissue function is revealed with a level of resolution that is not achieved by the use of single‐cell RNA‐seq in isolation. [4, 5]. However, a characterization relying solely on a single molecular modality is inherently limited, as it lacks the resolution to uncover the critical cross‐omics associations that bridge spatial organization, molecular expression, and functional phenotypes. The recognition of this limitation has spurred the development of spatial multi‐omics technologies, which profile multiple molecular layers (e.g., transcriptome and proteome) simultaneously from the same tissue section. Pioneering techniques such as DBiT‐seq [6], SM‐Omics [7], SPOTS [8], Stereo‐CITE‐seq [9], and 10x Genomics Xenium [10] now permit the coordinated measurement of RNA and dozens to hundreds of proteins. Notably, spatial‐CITE‐seq achieves the co‐indexing of whole transcriptomes with high‐plex (∼200–300) proteins, representing the highest spatial protein multiplexing achieved to date [11]. A key advantage of directly measuring proteins is their strong dose dependence and direct functional activity, which often correlates more directly with phenotype than transcript levels. With the development of these paired assays, the complex interplay between multi‐omics is effectively captured, revealing how interactions shape the biogenesis and physiological role of spatial domains. These processes are no longer viewed in isolation but as a unified regulatory network.

To fully harness the biological insights embedded within these datasets, a wide spectrum of computational strategies has been designed. Earlier strategies were primarily focused on the integration of disparate molecular layers to construct a unified representation of cellular states. Seurat's Weighted Nearest Neighbors (WNN) [12] utilizes a weighted strategy for this purpose, whereas probabilistic models such as totalVI and MultiVI [13, 14] are employed to model joint distributions across modalities. Simultaneously, a separate research branch was established to exploit the spatial context as a key factor in determining cellular identity. Specifically, graph neural networks (GNNs) are employed by methods such as STAGATE [15], MENDER [16], and SEDR [17] to aggregate information from neighboring spots. By accounting for these spatial dependencies, the transcriptomic data is utilized more effectively to define biological niches. In more recent developments, another suite of algorithms has emerged to tackle the full integration of spatially‐resolved multi‐omics data. Among these, SpatialGlue [18] advocates the complementary fusion of feature similarities and spatial neighbor information. This integration is achieved through a dual‐attention mechanism that adaptively aggregates information within and across omics layers. A dependency‐aware generative model is adopted by spaMultiVAE [19], which is specifically designed to capture spatial correlations across modalities. Alternative solutions are also provided by MISO and COSMOS. In MISO [20], a fusion mechanism is implemented by leveraging outer product calculations to refine the interaction between molecular layers. COSMOS [21] determines the relative contribution of each modality by assigning weights via the Seurat WNN algorithm.

Despite these technical advances, the problem of defining spatial domains is only partially addressed by existing efforts, which prioritize data reconciliation over biological insight, and the underlying cross‐omics regulatory mechanisms that drive the formation of tissue structures remain largely unexplored. It is true that molecular regulation is intrinsically linked to spatial context, since molecular interactions are typically shaped by a cell's spatial neighborhood and signaling environment [22, 23]. Spatial multi‐omics data is thus expected to provide essential cues for decoding this complex regulatory logic. However, several technical constraints expose vulnerabilities: transcriptomic data is often highly sparse [19], and protein measurements are prone to noise [24]. These issues can distort biological signals and undermine downstream analysis. This situation imposes a dual challenge upon the field, where the precise delineation of tissue organization is expected to couple with the inference of domain‐specific regulatory mechanisms from imperfect data.

Beyond spatial domain identification, decoding the molecular regulatory mechanisms that drive tissue organization remains a critical challenge. Recently, several computational tools have emerged to infer gene regulatory networks (GRNs) in single‐cell and spatial contexts. For example, SFINN [25] incorporates spatial location into a deep learning framework to augment cellular neighborhood networks for GRN inference. Similarly, multi‐omics approaches like SCRIPro [26] and CeSpGRN [27] have been proposed to reconstruct region‐specific GRNs by integrating transcriptomic profiles with spatial or epigenomic datasets. Despite these advances, existing regulation‐focused approaches exhibit notable limitations when applied to complex spatial multi‐omics data. First, they are largely constrained to single modalities (e.g., relying solely on transcriptomics) or depend heavily on predefined epigenomic reference motifs. This reliance makes them poorly equipped to dynamically learn unguided, cross‐omics (e.g., gene‐protein) regulatory affinities directly from the topological structure of the data. Second, spatial context is frequently treated as a mere distance‐based smoothing penalty, rather than being explicitly modeled alongside intra‐spot molecular interactions. As a result, these methods often fail to bridge the gap between macroscopic spatial architectures and their localized, multi‐layered regulatory drivers.

To tackle these limitations, we introduce SMOReg, a hierarchical graph‐based framework designed to bridge the gap between spatial domain discovery and the elucidation of interpretable regulatory mechanisms. SMOReg follows multi‐stage pipeline: at the intra‐spot level, it first refines multi‐omics signals through biologically informed graph convolutional networks, then uses a cross‐graph matching strategy to model multilayer molecular interactions, and finally employs a dual‐attention module to fuse these modalities into an enhanced representation for each spot. At the inter‐spot level, SMOReg builds dual‐context graphs informed by both spatial adjacency and feature similarity, and applies contrastive learning to obtain coherent domain delineation. By bridging these two scales, SMOReg not only improves spatial clustering performance but also pinpoints the critical regulatory interactions that drive domain‐level heterogeneity. SMOReg contributes three main innovations that overcome key limitations of prior approaches: (1) a hierarchical graph modeling scheme that directly deciphers spatial organization and its regulatory mechanisms; (2) a cross‐graph matching strategy that automatically learns domain‐specific, multilayer molecular affinities; and (3) dual‐context graph contrastive learning that uncovers previously unannotated functional microenvironments within tissues. We rigorously validated SMOReg on three real tissue slice datasets: human lymph node, human tonsil, and the mouse thymus slice. In all experiments, SMOReg is capable of capturing finer spatial domains alongside corresponding molecular regulatory mechanisms. For instance, in the human tonsil, SMOReg distinguished the light zone of the germinal center, marked by signature regulation involving *STAT3* and CD151, and the dark zone, characterized by regulation involving *VPREB3* and CD19.

## 2 Results

### 2.1 Motivation and Overview

**FIGURE 1:**
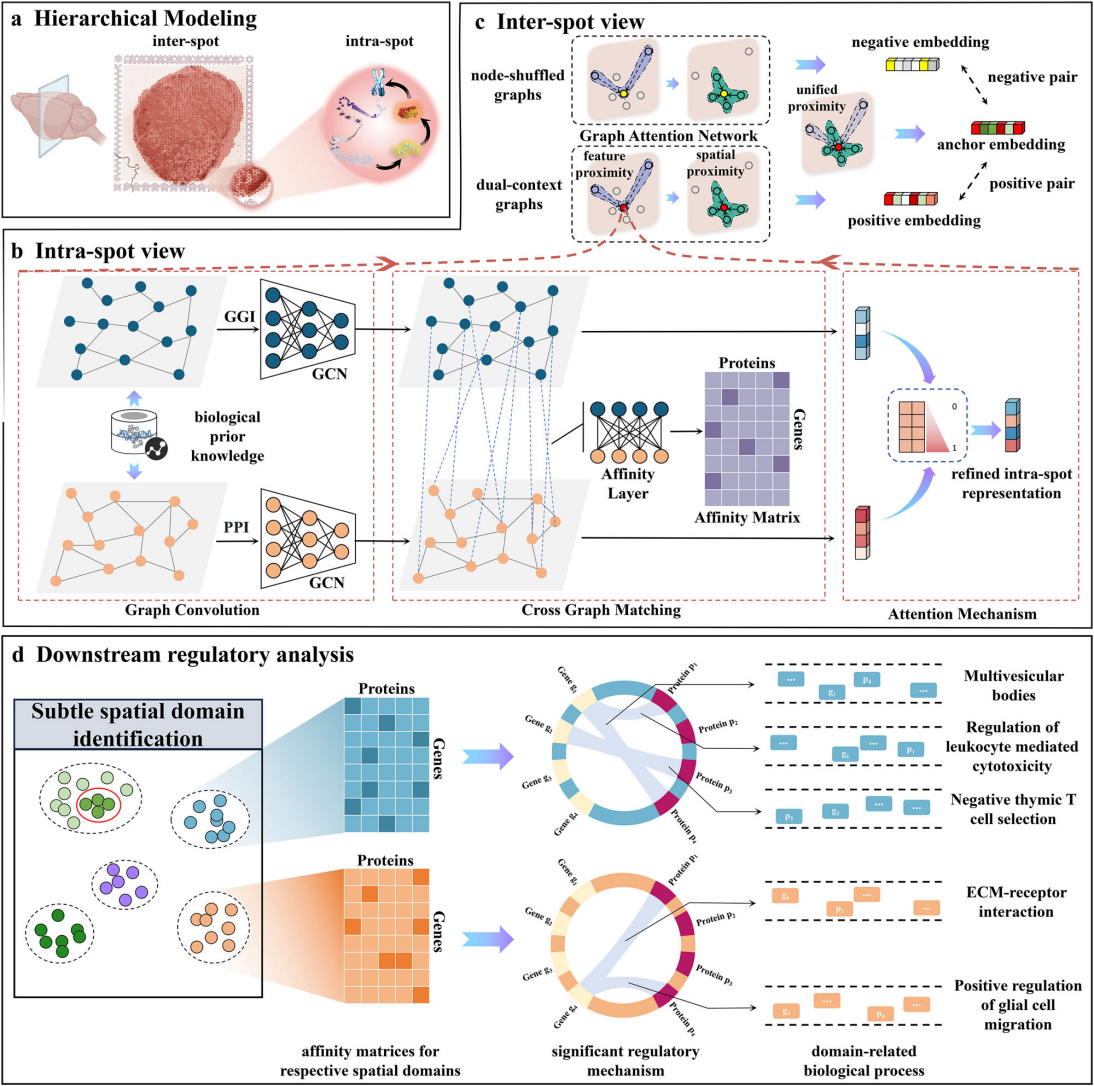
Overview of SMOReg. (a) Hierarchical modeling of spatial multi‐omics data. SMOReg processes paired transcriptomic and proteomic profiles from tissue sections through two complementary views: the intra‐spot level models molecular regulatory interactions within individual spots, while the inter‐spot level captures spatial dependencies across neighboring spots. This dual perspective integrates local molecular context with global tissue architecture. (b) Intra‐spot graph modeling. Gene–gene and protein–protein interaction graphs are constructed using expression‐derived node features and biologically curated edges. Graph convolutional networks encode intra‐omics context, followed by a cross‐graph matching module that learns gene–protein affinities. A dual‐attention mechanism then adaptively fuses both omics layers into refined spot‐level representations. (c) Inter‐spot representation learning via graph contrastive strategy. Spot embeddings from (b) serve as nodes in two context graphs: a spatial proximity graph (via physical closeness) and a feature proximity graph (via semantic closeness). A two‐layer graph attention network is trained on both graphs to generate positive embeddings. A readout function aggregates neighborhood features from the unified graph to obtain anchor embeddings. Negative samples are obtained by shuffling node features and reprocessing through the same GAT. Contrastive loss encourages alignment between anchor–positive pairs and separation from negatives. (d) Interpretable downstream analysis. Inter‐spot embeddings are clustered to identify spatial domains. Domain‐specific affinity matrices are used to extract key gene–protein regulatory pairs, visualized via chord diagrams and functionally interpreted through enrichment analysis.

SMOReg is designed to decode tissue organization by jointly modeling molecular regulation at cellular resolution and spatial context at tissue scale. As illustrated in Figure 1a, the framework analyzes a spatial multi‐omics dataset from two complementary perspectives: (1) an intra‐spot view, which models cross‐omics molecular interactions (e.g., protein‐gene relationships) within individual spots to infer cellular‐level regulatory programs; and (2) an inter‐spot view, which captures the influence of the tissue microenvironment and spatial context on cell states across neighboring spots. This hierarchical strategy enables SMOReg to bridge intracellular regulatory mechanisms with emergent tissue‐level phenotypes, thereby yielding spatial domains that are both data‐driven and biologically interpretable.

SMOReg implements this hierarchical graph modeling with dedicated modules, as shown in Figure 1b,c. Intra‐spot analysis starts the process. Gene‐gene interaction (GGI) graphs and protein–protein interaction (PPI) graphs are constructed from prior biological knowledge. Graph convolutional layers process these graphs, allowing information to propagate through the network structures. This propagation refines the node embeddings and captures regulatory dependencies within each omics layer. Next, a cross‐graph matching module takes over. It learns pairwise affinities between nodes from the two graphs, computed by a trainable affinity layer. The resulting affinities are assembled into a single unified matrix. Cross‐graph convolution is subsequently applied, enabling bidirectional message passing that allows each omics layer to progressively incorporate regulatory context from the other. SMOReg finally uses a dual‐attention mechanism to weight each omics layer, generating a refined representation for every spot. To capture inter‐spot relationships, the model employs a dual‐context graph contrastive learning strategy. We construct two complementary graphs: a spatial proximity graph for local physical neighborhoods and a feature proximity graph (via Seurat WNN [12]) for functional similarities across the tissue. By contrasting these two views, SMOReg learns embeddings that maintain both spatial continuity and functional homology.

To gain biological insights, SMOReg employs a downstream analytical pipeline as illustrated in Figure 1d. Once the model is trained, we identify spatial domains by clustering the inter‐spot embeddings. Thanks to SMOReg's hierarchical design, these embeddings capture subtle biological nuances, making it possible to resolve even fine‐grained tissue substructures. SMOReg further distils prominent gene–protein interactions for each spatial domain from its affinity matrices. By presenting these regulatory programs as chord diagrams, the model offers an intuitive snapshot of domain‐specific molecular cross‐talk. To ground these regulatory pairs in a functional context, we perform enrichment analysis to map them to specific biological processes. By bridging latent embeddings with mechanistic insights, this pipeline allows researchers to move beyond simple spatial mapping. It enables the interpretation of domain‐specific regulatory programs, which is crucial for understanding how molecular regulation defines cellular phenotypes in their native tissue environment.

### 2.2 Benchmarking SMOReg and Existing Methods on Simulated Data

**FIGURE 2:**
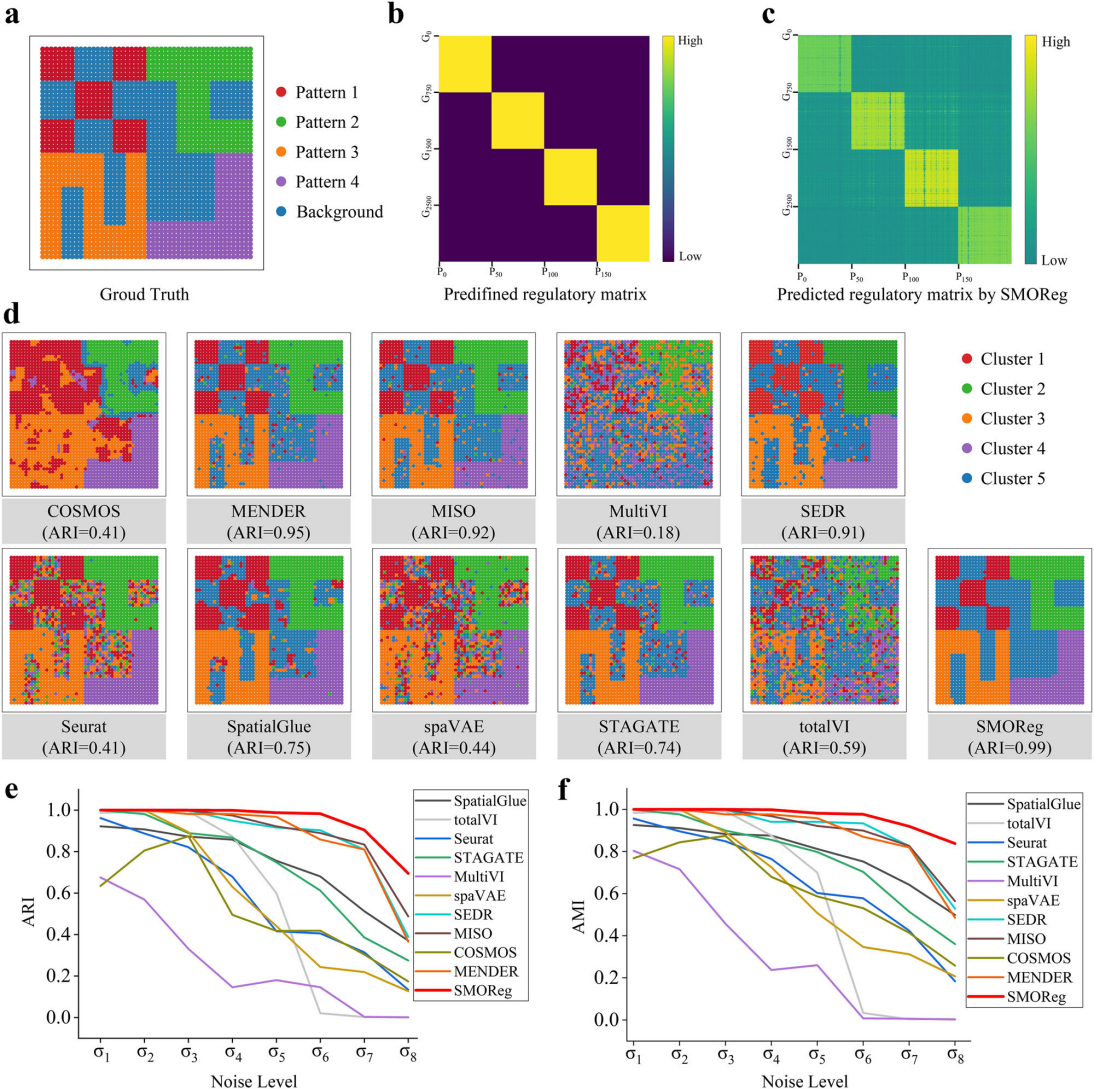
SMOReg robustly identifies spatial domains and reveals domain‐specific molecular regulatory interactions in simulated data. (a) Ground‐truth spatial domains used in simulations, comprising four expression patterns and one background region. (b) Binary matrix of predefined gene–protein regulatory relationships. Yellow blocks indicate domain‐specific regulatory regions between 3,000 genes (rows) and 200 proteins (columns). (c) Domain‐specific regulatory affinity matrix learned by SMOReg. Color intensity reflects the frequency of gene–protein interactions within each spatial pattern, with diagonal blocks corresponding to the four simulated patterns. (d) Spatial domain identification results under moderate noise ($\sigma_{5}$) by SMOReg and ten competing methods. (e, f) Performance trends of SMOReg and ten competing methods evaluated by ARI (e) and AMI (f) across increasing Gaussian noise levels. The noise levels on the x‐axis ($\sigma_{1}$ to $\sigma_{8}$) quantitatively correspond to an added Gaussian noise variance ranging from 50% to 200% of the mean expression value.

We first evaluated SMOReg on simulated spatial multi‐omics data to assess its ability to identify spatial domains and associated molecular regulatory relationships in a controlled setting. The simulated dataset comprised five spatial domains, including four with distinct expression patterns and one background cluster devoid of domain‐specific signals, designed to mimic regions lacking spatial coherence due to technical or biological noise. Each sample included a genomics layer with 3,000 genes sampled from a zero‐inflated negative binomial distribution to reflect real data sparsity, and a proteomics layer with 200 proteins simulated using a negative binomial distribution. To emulate measurement noise, Gaussian noise with zero mean and increasing variance ($\sigma_{1}$–$\sigma_{8}$) was added to both omics layers. These noise levels are denoted as $\sigma_{1}$ through $\sigma_{8}$. Specifically, this progression quantitatively corresponds to an added Gaussian noise variance ranging from 50% to 200% of the mean expression value for each molecule. Ground‐truth spatial domain labels (Figure ) and predefined inter‐omics regulatory associations (Figure 2b) were provided for validation. Further simulation details are available in Note S1. We compared SMOReg against ten representative methods spanning several categories: spatial multi‐omics integration (SpatialGlue, COSMOS, MISO, spaVAE), non‐spatial multi‐omics integration (Seurat, totalVI, MultiVI), and spatial transcriptomics‐based domain detection (STAGATE, SEDR, MENDER). For methods not originally supporting multi‐omics input, genomics and proteomics data were concatenated into a unified feature vector to ensure compatibility. To quantitatively estimate clustering performance against ground truth, we employed seven evaluated metrics: Adjusted Rand Index (ARI), Adjusted Mutual Information (AMI), Normalized Mutual Information (NMI), Homogeneity, Completeness, V‐measure, and Mutual Information.

We assessed the robustness of spatial domain identification under varying noise levels. Under a mid‐to‐high noise setting ($\sigma_{5}$), SMOReg accurately reconstructed all five spatial domains, with coherent structure and sharp boundaries closely matching the ground truth (Figure 2d). In contrast, COSMOS, totalVI, and MultiVI failed to recover specific patterns such as Pattern 1 and Pattern 3, while STAGATE, SpatialGlue, and MISO produced fragmented or blurred domain boundaries with limited discriminability between structured and background regions. To systematically quantify performance, we evaluated all methods across eight noise levels ($\sigma_{1}$–$\sigma_{8}$; Figure 2e,f). As noise increased, performance in ARI and AMI declined across all methods. Although several methods (totalVI, MENDER, MISO, SEDR) performed comparably to SMOReg under low noise ($\sigma_{1}$–$\sigma_{3}$), SMOReg demonstrated superior robustness at higher noise levels ($\sigma_{6}$–$\sigma_{8}$), maintaining the highest scores and most gradual performance decline. This consistent advantage was further corroborated by the other six evaluation metrics (Figure S1c), confirming SMOReg's stability and accuracy across a wide range of noise conditions. Notably, a defining advantage of SMOReg is its ability to jointly infer spatial domains and the underlying molecular regulatory interactions specific to each domain‐a capability not supported by any of the baseline methods examined. As illustrated in Figure 2c, the learned gene–protein regulatory associations for Patterns 1–4 are represented as a unified affinity matrix, where each element denotes the frequency of a regulatory pair observed within the corresponding domain. Four distinct diagonal blocks emerge in the matrix, each aligning precisely with one spatial pattern, reflecting strong domain‐specific regulatory architecture. SMOReg successfully recovered 91.06% of the ground‐truth regulatory links. We further evaluated its robustness to increasing noise levels in inferring regulatory relationships (Figure S1d). Although recovery rates gradually declined with higher noise, performance remained within a functionally informative range. Together, these results demonstrate that SMOReg effectively unifies spatial domain identification with the interpretation of domain‐specific molecular regulation within a single computational framework.

### 2.3 Identifying Spatial Domains and Regulatory Mapping of Immune Niches in the Human Lymph Node

**FIGURE 3:**
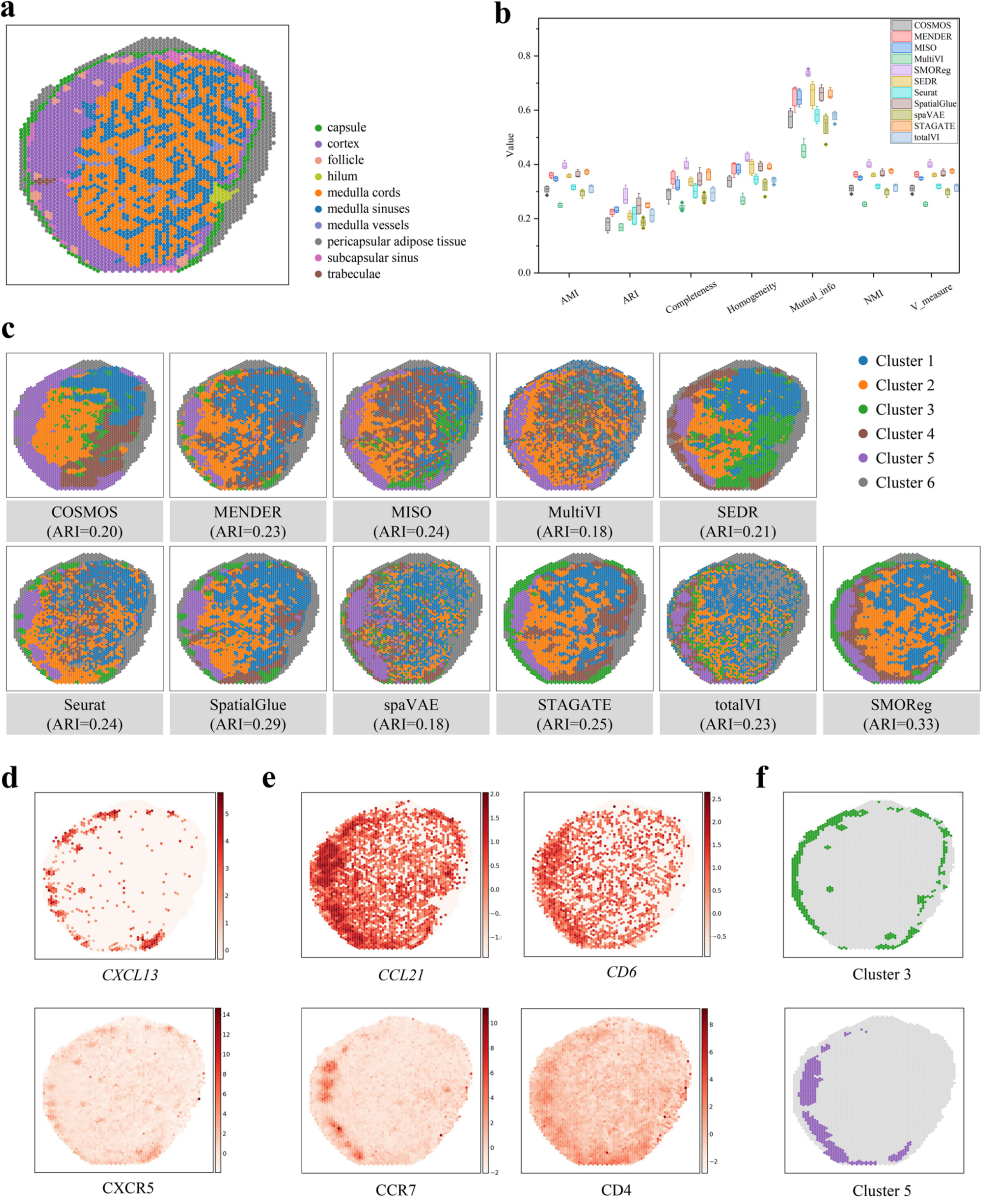
SMOReg accurately discovers the spatial domain and regulatory mapping of immune niches in the human lymph node. (a) Manual annotation of the lymph node tissue slice by SpatialGlue. (b) Box plots of seven evaluation metrics across all methods with the number of clusters ranging from 6 to 10. In the box plot, the center line denotes the median, box boundaries denote the upper and lower quartiles, and whiskers denote 1.5 times the interquartile range. (c) Spatial domain identification results from SMOReg and competing methods. Cluster colors are method‐specific and not directly comparable. (d) Spatial expression patterns of *CXCL13* and CXCR5 in SMOReg cluster 3. (e) Spatial expression patterns of *CCL21*, *CD6*, CCR7 and CD4 in SMOReg cluster 5. (f) Spatial plot of the capsule (cluster 3) and paracortex (cluster 5) identified by SMOReg.

In this section, we evaluated SMOReg on a human lymph node dataset with expert‐annotated anatomical structures, provided by the SpatialGlue team [18], which includes spatially resolved transcriptomic and proteomic profiles from the same tissue section. The annotated regions encompass major anatomical compartments: the outer pericapsular adipose tissue and capsule, and the inner cortex and medulla—the latter containing sinuses, cords, and vascular structures (Figure 3a). As shown in Figure 3c, while all methods successfully identified the pericapsular adipose region (cluster 6), the thin capsular structure was detected only incompletely by SpatialGlue, Seurat, and STAGATE, whereas SMOReg recovered it precisely (cluster 3). SMOReg also outperformed other methods in segmenting the cortex, a continuous anatomical region that was delineated as two cohesive subclusters (clusters 4 and 5) collectively covering nearly the entire cortical area and enveloping the medulla, consistent with the ground truth. Within the medulla (clusters 1 and 2), SMOReg revealed spatially organized and interwoven domain patterns, whereas other methods produced fragmented or disorganized clusters. Quantitative evaluation across multiple clustering metrics and a range of cluster numbers further confirmed that SMOReg consistently achieved the highest scores (Figure 3b) than other approaches, aligning with the qualitative improvements observed in the spatial visualizations.

Furthermore, differential expression analysis of both genomic and proteomic data revealed distinct molecular markers for each spatial domain identified by SMOReg (Figure S2a,b). Heatmap visualization confirmed strong concordance between these expression patterns and the inferred domains (Figures S3 and S4). Notably, SMOReg delineated a coherent subregion (cluster 5) within the annotated cortex, situated centrally and characterized by significant overexpression of the proteins CCR7 and CD4. Anatomically, the human lymph node cortex is divided into an outer cortex and a paracortex—the latter also referred to as the T‐cell zone, known for its abundance of CCR7 chemokines [28]. Spatial mapping of CCR7 and CD4 confirmed their co‐localization within this inner cortical region (Figure S5a–c), which aligned precisely with the extent of cluster 5 (Figure S5d). Evidence supporting this was also observed in hematoxylin and eosin (H&E)‐stained histological images from the Histology Guide website [29], which provides detailed, annotated microscopic slides of human tissues. Figure S6b depicts the overall lymph node, whereas Figure 6a,c present zoomed‐in views of the inner cortex and the outer cortex, respectively. These images further confirm the presence of the paracortex layer in the human lymph node. Together, these results demonstrate that SMOReg can reveal biologically meaningful spatial substructures not captured in original annotations, supported by both molecular profiling and established anatomical knowledge.

To further interpret the spatial domains from a regulatory perspective, we performed downstream analysis of cross‐omics interactions inferred by SMOReg for each spatial cluster (Note S2). Although the number of available proteins in this human lymph node dataset limited the scope of detectable regulatory mechanisms, SMOReg successfully identified key gene–protein interactions in clusters 3, 5, and 6 (Figure S2c) and enriched relevant biological terms for each domain (Figure S2d). In cluster 3, the *CXCL13*–CXCR5 pair was associated with extrafollicular and follicular B cell activation. In the paracortex (cluster 5), the *CCL21*–CCR7 interaction was linked to T cell chemokine receptor activity, while *CCL21*–CD4 correlated with positive regulation of the ERK1/2 cascade. In the pericapsular adipose region (cluster 6), CD22, together with *FABP4* and CD19, connected lipid metabolism to immune regulation near the follicular capsule. These pathways are supported by multiple lines of evidence (Note S3). Notably, many molecules involved in these regulatory interactions overlapped with differentially expressed markers (Figure 3d, e), indicating that SMOReg prioritizes functionally relevant molecules within each domain. By integrating enrichment results, prior knowledge, and expression data, SMOReg accurately delineated the complete structure of the capsule (cluster 3) and paracortex (cluster 5) in human lymph node tissue (Figure 3f), outperforming all other benchmark methods.

### 2.4 Capturing Anatomical Structures and Regulatory Landscape of Germinal Center Reaction in the Human Tonsil

**FIGURE 4:**
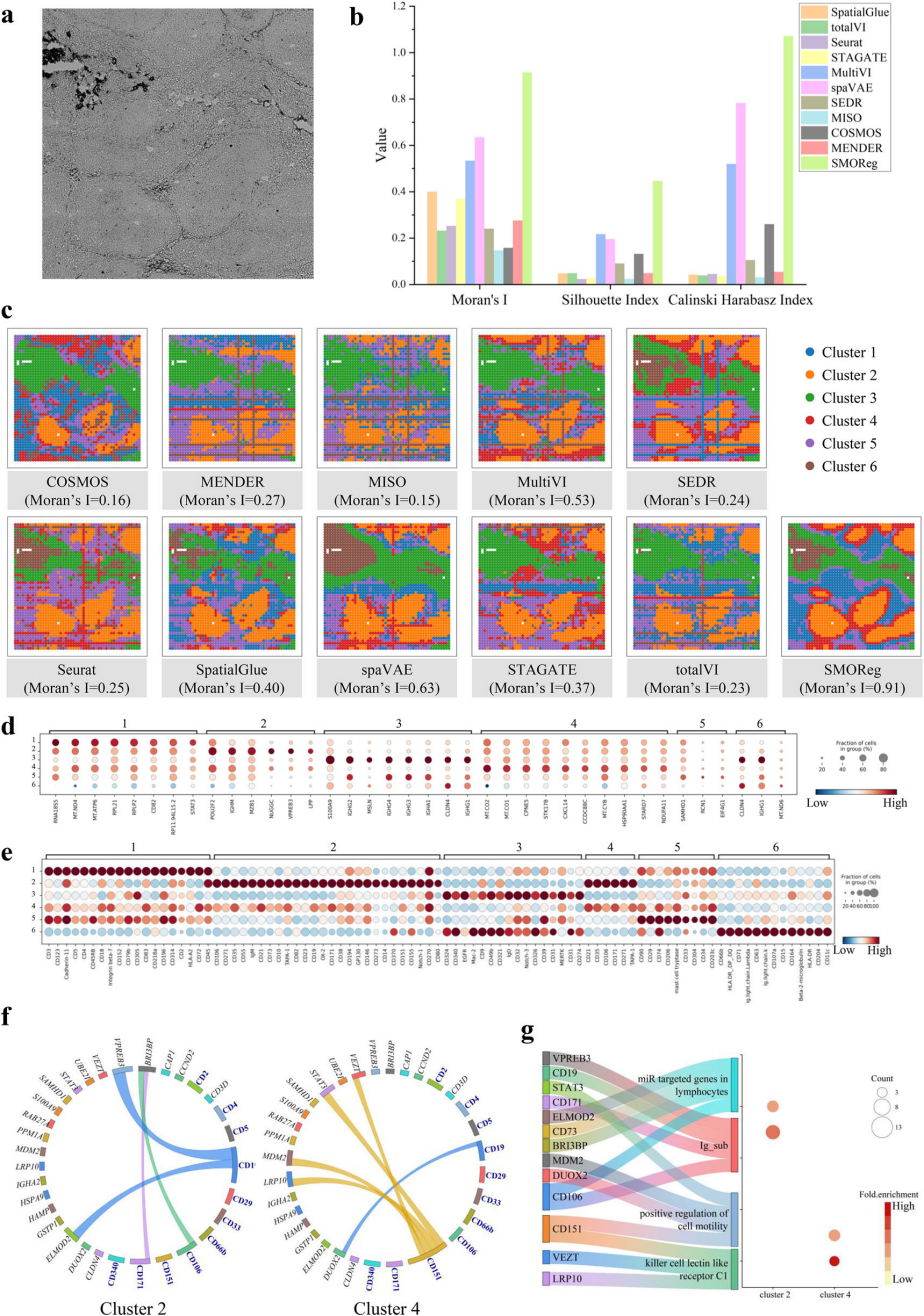
SMOReg captures anatomical structures and the regulatory landscape of germinal centre reaction in the human tonsil. (a) High‐resolution microscope image of the human tonsil section. (b) Comparative performance of SMOReg and ten baselines across three unsupervised metrics: Moran's I, Silhouette Index, and Calinski–Harabasz Index. Higher values reflect better clustering quality. (c) Spatial domain mapping by SMOReg and competing methods. Cluster colors are method‐specific and not directly comparable. (d) Dot plot of transcriptomic differential expression across SMOReg clusters. Dot size represents the fraction of expressing cells; color indicates average expression. (e) Dot plot of proteomic differential expression across SMOReg clusters. (f) Chord diagrams of gene–protein regulatory interactions in clusters 2 and 4. Nodes represent molecules (genes in italics, proteins in upright font), and chords denote high‐affinity regulatory pairs. All diagrams share the same node set and color scheme. (g) Sankey‐bubble plot linking enriched biological processes (right) to regulatory molecules (left). Bubble color and size encode fold enrichment and number of involved molecules, respectively.

We next applied SMOReg to a human tonsil spatial multi‐omics dataset generated by spatial CITE‐seq [11], which simultaneously profiles the whole transcriptome and 273 human protein markers across a 2.5 mm × 2.5 mm tissue region, shown in high‐resolution microscopy image in Figure 4a. We compared SMOReg against ten benchmark methods (Figure 4c) and observed that several methods (e.g., MENDER, MISO, SEDR) introduced stripe‐like artifacts originating from transcriptomic noise, whereas SMOReg, by integrating both omics layers with prior biological knowledge, produced spatially coherent domains with effectively suppressed technical bias. The domains identified by SMOReg closely matched those reported in the original spatial CITE‐seq study but exhibited improved smoothness and spatial continuity. Based on this reference, we annotated the six SMOReg clusters as: T cell zone (cluster 1), germinal center dark zone (cluster 2), crypt epithelial region (cluster 3), germinal center light zone (cluster 4), extrafollicular region (cluster 5), and peripheral blood cell region (cluster 6). Although the peripheral blood cell region (cluster 6) was identified by multiple methods including SMOReg, SEDR, Seurat, SpatialGlue, and spaVAE, SMOReg again demonstrated superior resolution by distinctly separating the germinal center light zone (cluster 4) and dark zone (cluster 2)—a functionally critical anatomical subdivision that was largely indistinguishable in other methods. This computational delineation is histologically corroborated in H&E‐stained sections of human tonsil [30] (Figure S8a,b), where the germinal center is visibly partitioned into a densely cellular outer layer (light zone) and a less compact central region (dark zone). These observations confirm that the germinal center indeed comprises a two‐layered structure at the anatomical level. In the absence of histological annotations, we quantitatively evaluated clustering quality using three unsupervised metrics: Moran's I (spatial autocorrelation), Silhouette Index (cluster compactness), and Calinski–Harabasz Index (inter‐cluster separation). SMOReg consistently achieved the highest scores across all three metrics (Figure 4b), underscoring its superiority in identifying subtle yet biologically meaningful spatial structures. Specifically, while Moran's I incorporates physical spatial coordinates to construct the spatial weight matrix for assessing global spatial autocorrelation, both the Silhouette Index and the Calinski‐Harabasz Index were computed directly on the final high‐dimensional latent embeddings extracted by SMOReg. This approach rigorously evaluates the intrinsic cluster compactness and inter‐cluster separation within the learned feature space.

Differential expression analysis of both genomic and proteomic data identified key molecular markers associated with each spatial domain (Figure 4d,e; Figures S9 and S10). Notably, the two omics layers exhibited complementary patterns across several clusters. In cluster 3, differentially expressed proteins were detected in both clusters 3 and 6, whereas differentially expressed genes were predominantly concentrated in cluster 3. Similarly, while no significantly different genes were found in cluster 5, multiple proteins showed strong domain specificity. Complementary expression was also observed in clusters 4 and 6. These patterns highlight SMOReg's ability to effectively integrate informative signals from both omics layers, ensuring robust domain identification even when one modality contains noisy or uninformative features.

We next investigated the regulatory mechanisms underlying key spatial domains in the human tonsil, focusing on the germinal center (GC) light and dark zones'critical microstructures where B cells undergo proliferation, somatic hypermutation (SHM), and affinity‐based selection during T cell–dependent immune responses [31]. Cross‐omics regulatory interactions and associated biological processes for each domain are visualized in Figure 4f,g, and Figure S11.

In the GC dark zone (cluster 2), where B cells expand and diversify via SHM, SMOReg identified a regulatory interaction between *VPREB3* and CD19 (key molecular markers in Figure 4d, e), assigned to the immunoglobulin subunit (*Ig_sub*) functional module. This complex forms part of the structural backbone of the B cell receptor (BCR). Both molecules are known participants in pre‐BCR–mediated signaling [32] and are functionally linked in the STRING database [33]. The pre‐BCR coordinates early B cell expansion and initiates light chain recombination and SHM that takes place later in the GC dark zone function [34].

In the GC light zone (cluster 4), where B cells from the dark zone undergo affinity‐based selection, SMOReg detected an interaction between *STAT3* and CD151, enriched for positive regulation of cell motility. Both molecules facilitate B cell migration: *STAT1* (a STAT family member related to *STAT3*) reduces cell adhesion [35] and participates in inflammatory signaling guiding marginal zone B cell differentiation [36], while CD151 enhances motility by regulating integrin trafficking [37]. Transcriptional evidence from GeneCards [38] further supports a direct regulatory relationship between them, underscoring their role in preparing B cells for affinity selection within the GC light zone.

The regulatory landscape uncovered by SMOReg captures a coordinated molecular cascade reflecting the initiation of the germinal center reaction, delineating a spatially resolved, multi‐omics view of cellular trafficking, immune regulation, and developmental dynamics. The germinal center dark and light zones represent subtle spatial domains that are often challenging to delineate with existing methods. SMOReg, however, not only distinguishes these structurally subtle regions but also elucidates their underlying cross‐omics regulatory interactions, providing a mechanistically interpretable and refined perspective on these complex tissue compartments.

### 2.5 Deciphering Layered Tissue Compartments and Distinct Regulatory Programs in the Mouse Thymus

**FIGURE 5:**
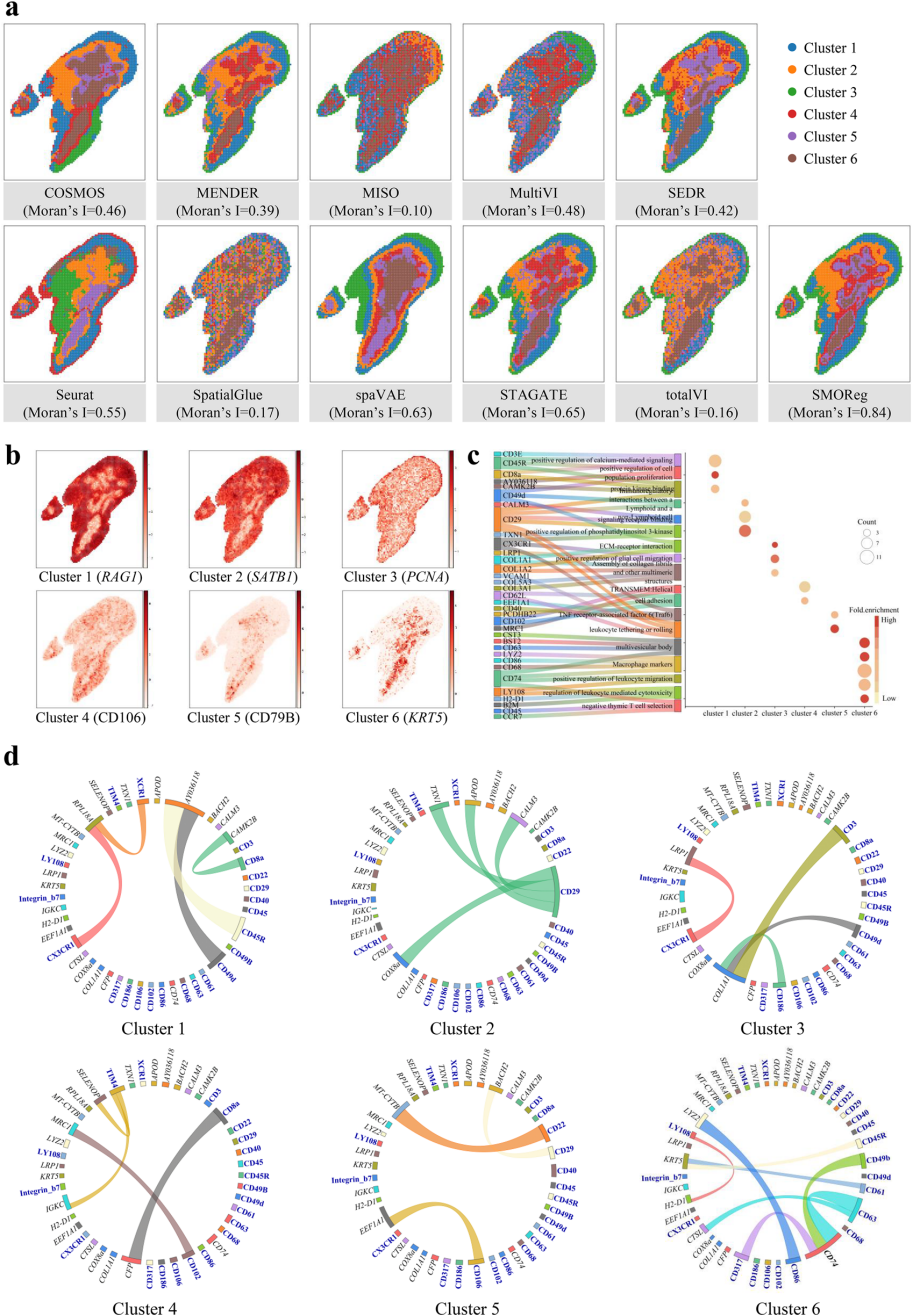
SMOReg deciphers layered tissue compartments and distinct regulatory programs in the mouse thymus. (a) Spatial domain mapping of mouse thymus by SMOReg and competing methods. Cluster colors are method‐specific and not directly comparable. (b) Spatial expression patterns of representative markers across SMOReg clusters 1–6. Genes are italicized (e.g., *RAG1, SATB1, PCNA, KRT5*); proteins are upright (e.g., CD106, CD79B). (c) Sankey‐bubble plot linking enriched biological processes to regulatory molecules. Left: molecular contributors; right: enriched terms with bubble color/size indicating fold enrichment and molecule count. (d) Chord diagrams of SMOReg‐inferred regulatory interactions. Nodes (genes in italics, proteins upright) represent molecules; chords denote high‐affinity pairs. All diagrams share the same node set and color scheme.

We next evaluated SMOReg on a newly generated mouse thymus dataset acquired via Stereo‐CITE‐seq [9], which provides spatially resolved co‐measurement of mRNAs and proteins at subcellular resolution. On this tissue section, SMOReg again produced spatially coherent domain delineations (Figure 5a) and achieved superior scores on unsupervised clustering metrics (Figure S12g), demonstrating a consistent advantage over competing methods in identifying biologically meaningful tissue compartments.

In the absence of histological annotations, we annotated spatial domains by mapping known RNA and protein markers to each cluster identified using SMOReg (Figure 5b). The outermost layer (cluster 3) aligns with strong *PCNA* expression—a marker of proliferating double‐negative (DN) thymocytes in the subcapsular zone, which serves as a proliferative niche during early T‐cell development [39]. Adjacent to this region, cluster 1 shows high expression of double‐positive (DP) stage markers *TRBC2* and *RAG1* [39], along with co‐enrichment of the CD4 and CD8 proteins (Figures S12 and S13), supporting its identity as the outer cortex where $CD4^{+}$ and $CD8^{+}$ DP thymocytes reside. The inner cortex (cluster 2), marked by *SATB1* [39], lies deeper and corresponds to a more mature DP stage [40]. SMOReg also cleanly isolated the thymic nurse cell area (cluster 4), with both *VCAM1* transcript and its protein product CD106 tracing its boundary—a microenvironment where long‐lived DP thymocytes undergo positive selection [41]. The cortico‐medullary junction (cluster 5) spatially coincides with CD79B expression, while the medulla (cluster 6) is marked by multiple identifiers, including *KRT5* (mTEC‐specific), *CCR7*, and CD40 [41, 42, 43, 44].

SMOReg accurately reconstructed the anatomical layers of the thymus, spanning from the outer capsule to the inner medulla, and produced a spatial map that highly correlates with established molecular markers. By contrast, alternative methods such as MISO and totalVI performed poorly on this dataset, yielding fragmented patterns characterized by spurious signals and scattered noise. Notably, while most models could identify broad regions like the medulla, they consistently failed to resolve finer substructures, such as the capsular zone (cluster 3) and the cortico‐medullary junction (cluster 4). This technical limitation is significant, as it may overlook the specialized immunological microenvironments required for T cell development. The biological validity of the SMOReg‐defined domains was further confirmed through differential expression analysis (Figure S12 and S13). The distinct spatial distributions of the identified marker genes and proteins not only delineate clear boundaries but also reinforce the functional relevance of each tissue compartment (Figure S14a,b).

By leveraging SMOReg's ability to pinpoint domain‐specific molecular interactions, we extracted unique gene–protein regulatory links and their corresponding biological pathways for each spatial compartment (Figure 5c,d). To illustrate the functional significance of these findings, we highlight the capsular zone and the medulla as case studies. These two regions were accurately delineated by SMOReg and serve to clarify the key regulatory mechanisms that drive their specialized roles within the anatomical organization of the thymus.

In the capsular zone (cluster 3), the expression of *COL1A1* and CD49d was associated with the KEGG ECM (Extracellular Matrix) ‐ receptor interaction pathway (Figure 5c; Figure S14c). As a primary structural component of the thymic capsule, type I collagen (encoded by *COL1A1*) is secreted by fibroblasts to establish a fibrous scaffold for progenitor cell adhesion. This process is complemented by CD49d, an integrin subunit encoded by *ITGA4*, which binds to ECM ligands to facilitate cellular attachment. Beyond its anchoring function, this integrin–ECM interaction plays a pivotal role in transducing signals that regulate cytoskeletal remodeling, mechanical tension, and matrix assembly. These mechanisms are essential for maintaining structural integrity and enabling the dynamic remodeling of the capsular microenvironment [45]. Such findings are further supported by functional annotations related to the Assembly of collagen fibrils and other multimeric structures and *Positive regulation of glial cell migration*, both of which underscore the importance of ECM organization in this specific region.

In the medulla (cluster 6), the enrichment of the term “regulation of leukocyte mediated cytotoxicity” reflects the crucial role of this compartment in refining T cell effector functions. A regulatory axis that integrates antigen presentation with T cell receptor signaling is suggested by the coordinated involvement of *H2‐D1*, a classical MHC class I molecule, and LY108, a SLAM family receptor. This mechanism shapes the cytotoxic potential of developing $CD8^{+}$ SP T cells while simultaneously enforcing central tolerance. Such observations align with the established functions of medullary thymic epithelial cells (mTECs) in negative selection and effector differentiation [41, 46]. Furthermore, the co‐enrichment of *CD74* and CD63 within the multivesicular bodies (MVBs) pathway highlights the presence of active antigen‐processing machinery. As the invariant chain of MHC class II, *CD74* is processed inside MVBs to facilitate peptide loading, whereas CD63 serves as a canonical marker for these organelles [47, 48]. The spatial co‐occurrence of these molecules implies robust antigen‐loading activity, which is essential for central tolerance. Ultimately, these findings underscore the functional specialization of the medulla in presenting self‐antigens to maturing T cells.

Collectively, these findings highlight the effectiveness and interpretability of SMOReg in identifying spatial domains through the integrated analysis of molecular signatures, regulatory mechanisms, and anatomical structures. By capturing spatial heterogeneity from the dual perspectives of the tissue microenvironment and cross‐omics regulatory relationships, SMOReg facilitates the precise delineation of fine‐grained tissue compartments. Furthermore, the model reveals the underlying multilayer molecular programs that govern these regions, even within previously uncharacterized tissue contexts.

### 2.6 Ablation Study and Sensitivity Analysis Experiments

To rigorously validate the structural advantage of the cross‐graph matching module (a core methodological innovation of our framework) and the overall stability of SMOReg, we conducted a comprehensive ablation study followed by a hyperparameter sensitivity analysis. First, we compared the full SMOReg model against a baseline (namely SMOReg_wo_cross_matching) where the cross‐graph convolution step was bypassed, feeding the refined intra‐omics features directly into the dual‐attention fusion module. As detailed in Note S4 and Figures S15 and S16, the ablation results confirm that the cross‐graph matching mechanism provides significant quantitative performance gains and enhanced stability. More importantly, it is indispensable for delineating fine‐grained, biologically meaningful spatial domains, ensuring that critical anatomical structures are accurately resolved without losing essential biological insights for downstream regulatory analysis.

Beyond structural integrity, the framework's overall reliability also depends on its robustness to initial graph‐building settings. To address this, we systematically evaluated SMOReg's sensitivity to the number of Weighted Nearest Neighbors (k). As detailed in Note S5 and illustrated in Figure S17, the spatial domain identification performance remains highly stable across a reasonable operational range. This analysis confirms that SMOReg successfully leverages the WNN algorithm to reliably capture local multimodal signals without being overly sensitive to precise parameter tuning, thereby ensuring the robustness of our downstream clustering results.

## 3 Discussion

We introduce SMOReg, a hierarchical graph‐based framework designed to integrate spatial multi‐omics data with interpretable regulatory modeling. This approach facilitates the joint identification of tissue spatial domains and their associated molecular interactions. By synthesizing both intra‐spot and inter‐spot perspectives, SMOReg effectively captures localized molecular activity alongside tissue‐level structural organization. At the intra‐spot level, the framework constructs molecular interaction graphs informed by prior biological knowledge. Through cross‐graph matching, it infers gene–protein regulatory affinities, thereby enabling the discovery of domain‐specific cross‐omics relationships. Such interactions are frequently overlooked by existing multi‐omics integration methods, highlighting SMOReg's capacity to resolve complex biological interdependencies.

Notably, SMOReg does not aim to map all possible regulatory links exhaustively. Instead, the framework prioritizes salient and spatially consistent interactions, which enhances biological plausibility while mitigating the risk of overfitting. Methodologically, a dual‐view learning strategy is employed: the inter‐spot module utilizes contrastive learning to guide Graph Attention Network (GAT) training, whereas the intra‐spot affinity layer is refined via top‐down feedback from global embeddings. This weakly supervised approach operates independently of manual labels or predefined gene–protein pairs, relying solely on structural and relational cues. By circumventing predefined cross‐omics constraints, SMOReg infers data‐driven regulatory relationships that reflect intrinsic, domain‐specific signals. This adaptability represents a core strength of the framework, allowing for the discovery of regulatory patterns directly from the data.

Another significant advantage of SMOReg is its inherent interpretability. The regulatory pairs identified by the model consistently concur with established pathways and anatomical contexts, a fact demonstrated through KEGG enrichment and case studies across lymph node, tonsil, and thymus tissues. Such alignment not only validates the model's predictions but also provides a robust foundation for generating new hypotheses regarding tissue organization and intercellular communication. Crucially, spatial heterogeneity cannot be ascribed solely to expression differences within a single omics layer. Our results indicate that domain boundaries often derive from coordinated cross‐omics behavior. Notable examples include the *CCL21*–CCR7 pair in the lymph node paracortex, as well as the *VPREB3*–CD19 and *STAT3*–CD151 interactions that distinguish germinal center zones in the tonsil. While models based purely on expression similarity may overlook this regulatory complexity, SMOReg explicitly incorporates both intra‐omics variation and inter‐omics interactions. Consequently, the framework yields spatial domains that are more biologically meaningful and easier to interpret.

Finally, it is important to acknowledge the boundary between computational predictions and physical ground truth. Although the domain‐specific gene‐protein interactions identified by SMOReg are strongly supported by functional enrichment analysis and existing literature, they fundamentally represent data‐driven, in silico hypotheses. To transition these predictions into definitive biological conclusions, future studies will benefit from orthogonal experimental validation. Targeted functional assays or spatial perturbation screens could be employed to physically verify these spatially resolved regulatory pairs, thereby further solidifying the mechanistic insights generated by our framework.

In summary, SMOReg provides a principled and interpretable framework for spatial multi‐omics analysis, facilitating both accurate domain discovery and mechanistic insights into domain‐specific regulation. By integrating structured biological knowledge with a hierarchical graph‐based model, the approach offers a versatile platform for deciphering how complex molecular interactions shape tissue architecture.

## 4 Methods

### 4.1 Data Description

#### 4.1.1 Human Lymph Node Dataset

We analyzed a human lymph node section (sample A1) from Long et al. [18], which was processed as a 5 μm FFPE section and profiled using the 10x Genomics CytAssist Visium platform. The transcriptome was captured using the Transcriptome Probe Set v2.0, and proteins were measured via a 35‐plex Human FFPE Immune Profiling Panel. Expert manual annotations based on H&E staining served as ground truth. The dataset includes 3,484 spots, 18,085 genes, and 31 proteins. Genes detected in fewer than ten spots were removed, and the remaining transcriptome data were normalized, log‐transformed, and standardized using SCANPY [49], with the top 3,000 highly variable genes (HVGs) retained. Protein data were centered log‐ratio normalized and standardized without further filtering.

#### 4.1.2 Human Tonsil Dataset

The human tonsil dataset was generated by Liu et al. [11] using spatial‐CITE‐seq, enabling co‐profiling of the whole transcriptome and 200–300 antibody‐derived tags (ADTs) at cellular resolution. Tissue sections were fixed, labeled with ADTs, and spatially barcoded on a microfluidic chip. No ground‐truth annotations were publicly available. We analyzed a subset containing 2,492 spots, 28,417 genes, and 273 proteins. Preprocessing matched that of the lymph node data, retaining all proteins.

#### 4.1.3 Mouse Thymus Dataset

A mouse thymus section was profiled using Stereo‐CITE‐seq [9] (BGI Genomics). The tissue came from a 6–8‐week‐old male C57BL/6 mouse, was fresh‐frozen in OCT, and sectioned at 10 μm. The sample was fixed with PFA, stained with DAPI, and permeabilized for 14 min before being applied to a 1 cm × 1 cm Stereo‐seq chip. The dataset included 27,445 genes and 128 targeted proteins. After removing genes expressed in fewer than ten spots, transcriptome data were normalized and standardized, and the top 3,000 HVGs were selected. Non‐specific isotype controls and poorly annotated proteins were excluded, yielding a final set of 3,393 spots, 3,000 genes, and 106 proteins.

Notably, no upfront data imputation was applied to the transcriptomic or proteomic profiles during the preprocessing of any real‐world datasets. To address the inherent sparsity and dropout noise typical of spatial omics data, SMOReg relies entirely on the subsequent biological prior‐informed message‐passing within the GCN encoders. By aggregating features across established molecular pathways, this graph‐based smoothing naturally mitigates technical noise without introducing the artificial biases often associated with explicit imputation algorithms.

### 4.2 Intra‐Spot Graph Modeling

#### 4.2.1 Construction of Molecular Graphs Based on Biological Priors

We constructed intra‐spot molecular graphs by incorporating prior regulatory knowledge to establish a gene–gene interaction (GGI) graph and a protein–protein interaction (PPI) graph for each spot. Consider a tissue slice with N spots, where each spot contained measurements for $N_{g}$ highly variable genes (HVGs) and $N_{p}$ proteins. An undirected molecular graph is defined as $G=\left(V,E\right)$, where $V\in R^{q\times 1}$ denotes the set of q molecules and E represents the set of connected edges between them. The adjacent matrix $A\in R^{q\times q}$ decodes the graph structure, with A(i,j)=1 if i‐th and j‐th molecules are correlated according to biological priors, otherwise 0.

To build the GGI graph, we derived functional associations from Gene Ontology (GO)  [50]. Each gene was mapped to its associated biological processes, and the functional similarity between two genes $g_{i}$ and $g_{j}$ is quantified using the Jaccard index:

(1) $$J_{i,j}=\frac{|N_{i}\cap N_{j}|}{|N_{i}\cup N_{j}|},$$

where $N_{i}$ and $N_{j}$ denote the sets of GO terms associated with $g_{i}$ and $g_{j}$, respectively. An edge is established between $g_{i}$ and $g_{j}$ if $J_{i,j}>\tau$, where τ is a predefined similarity threshold. For the PPI graph, we extracted protein–protein interactions from the STRING database  [33] with a confidence score threshold of 0.9.

#### 4.2.2 Data Refinement with Graph Convolutional Encoder

We employed standard Graph Convolutional Networks (GCNs) as encoders to refine omics profiles by integrating prior regulatory knowledge at the intra‐spot level. The computational intuition behind selecting a GCN for this specific task was rooted in the deterministic nature of the constructed graphs. Since the gene‐gene and protein‐protein interaction edges were derived from curated and reliable biological databases, a standard GCN was sufficient and efficient for propagating features smoothly across these established molecular pathways. This straightforward propagation refined the node embeddings without the computational overhead of dynamic edge re‐weighting. To preserve molecular heterogeneity while enabling knowledge sharing, each node was initialized with a d‐dimensional learnable embedding derived from its original expression profile, an approach shown to enhance feature diversity and encoding capacity  [51].

For each omics modality, a dedicated GCN encoder was applied to learn the latent representation $h_{i}$ for i‐th molecule. The representation at the l‐th layer is computed as:

(2) $$h_{i}^{l}=\sigma \left(\tilde{A}h_{i}^{l-1}W^{l-1}+b^{l-1}\right)$$

where $\tilde{A}=D^{-\frac{1}{2}}AD^{-\frac{1}{2}}$ denotes the symmetrically normalized adjacency matrix, D is the diagonal degree matrix with entries $D_{ii}=\sum_{j=1}^{N}A_{ij}$. W and b are the trainable weight matrix and bias vector, respectively, and σ(·) is a nonlinear activation function (ReLU). The input representation $h_{i}^{0}$ corresponds to the preprocessed one‐dimensional node feature. In practice, we used a single GCN layer (L=1), which suffices to capture informative signals from the local neighborhood.

#### 4.2.3 Affinity Learning with Cross‐Graph Matching Module

To learn domain‐specific regulatory relationships between genes and proteins in a data‐driven manner, we introduced a cross‐graph matching module that encoded inter‐omics interactions into a pairwise affinity matrix. To quantitatively estimate the interaction strengths between nodes from the GGI and PPI graphs, we explicitly formulated the affinity function $f_{aff}$ as a parameterized bilinear transformation. Let $h_{i}^{g}\in R^{d}$ and $h_{j}^{p}\in R^{d}$ denote the d‐dimensional representations of the i‐th node in the GGI graph and the j‐th node in the PPI graph, respectively. The affinity score between them is computed as follows:

(3) $$B_{i,j}=\sigma \left({\left(h_{i}^{g}\right)}^{T}W_{aff}h_{j}^{p}\right),\;i\in V_{g},\;j\in V_{p}$$

where $W_{aff}\in R^{d\times d}$ is the trainable weight matrix of the affinity layer. This bilinear formulation effectively captures the complex cross‐omics interaction dynamics by projecting the two distinct modality spaces into a shared correlation space. The sigmoid function σ(·) constrains the output values to the range (0, 1), ensuring numerical stability during training and yielding the final affinity matrix $B\in R^{N_{g}\times N_{p}}$. Each entry $B_{i,j}$ rigorously represented the learned regulatory interaction strength between the i‐th gene and the j‐th protein. A detailed architectural schematic mapping Equations (3) through (5) is provided in Figure S18 to explicitly illustrate the entire data flow, covering the generation of the affinity matrix, the argmax‐based node selection, and the subsequent feature concatenation.

To enable bidirectional information flow between omics layers, we implemented a cross‐graph convolution mechanism that propagates regulatory signals using the learned affinity matrix B. For each gene node, we selected the most relevant protein node via row‐wise argmax over B, and symmetrically selected the most relevant gene node for each protein node via column‐wise argmax. Features from these top‐affinity cross‐omics nodes were then transformed and concatenated with the original node representations. Formally, the cross‐graph update is defined as:

(4) $$\begin{array}{cc} {\tilde{h}}_{i}^{g} & =f_{\text{update}}\left([\;h_{i}^{g}\;∥\;f_{\text{cross}\_\text{conv}}\left(h_{j^{*}}^{p}\right)\;]\right),\;j^{*}=\arg\max_{j}B_{i,j} \end{array}$$

(5) $$\begin{array}{cc} {\tilde{h}}_{j}^{p} & =f_{\text{update}}\left([\;h_{j}^{p}\;∥\;f_{\text{cross}\_\text{conv}}\left(h_{i^{*}}^{g}\right)\;]\right),\;i^{*}=\arg\max_{i}B_{i,j} \end{array}$$

where $h_{i}^{g}$ and $h_{j}^{p}$ denote input representations of gene and protein nodes, respectively. The function $f_{\text{cross}\_\text{conv}}\left(\cdot \right)$ performs feature transformation across graphs, and [·∥·] denotes vector concatenation. The update function $f_{\text{update}}\left(\cdot \right)$ ‘implemented as a linear layer ’ projects the concatenated features back to the original dimensionality d. Let ${\tilde{H}}^{g}\in R^{N_{g}\times d}$ and ${\tilde{H}}^{p}\in R^{N_{p}\times d}$ denote the resulting cross‐omics representations for the GGI and PPI graphs, respectively. A detailed architectural schematic mapping Equations (3) through (5) is provided in Figure 18 to explicitly illustrate the entire data flow, covering the generation of the affinity matrix, the argmax‐based node selection, and the subsequent feature concatenation.

This design enabled a two‐level integration: at the intra‐omics level, GCNs propagate information within each molecular graph using biological priors; at the inter‐omics level, the cross‐graph convolution simulated regulatory interplay by allowing nodes in one omics layer to interact with highly associated nodes in another, thereby modeling mutual influence across molecular types.

#### 4.2.4 Attention Mechanism for Multi‐Omics Fusion

To integrate node representations derived from the GGI and PPI graphs, we introduced an attention‐based fusion module that adaptively weights the contribution of each omics modality. First, a graph‐level embedding $p^{m}$ for each molecular graph $G^{m}$ is obtained via a linear projection:

(6) $$p^{m}=W_{m}\left(\frac{1}{d}\sum_{i=1}^{d}{\tilde{H}}^{m}\left(i\right)\right)+b_{m}$$

where ${\tilde{H}}^{m}\left(i\right)$ represent the i‐th column of the refined node representation matrix ${\tilde{H}}^{m}$. $W_{m}$ and $b_{m}$ are the learnable parameters. The importance score $c^{m}$ for m‐th omics is computed as:

(7) $$c^{m}=\nu^{T}\cdot \tanh\left(W_{atten}p^{m}+b_{atten}\right)$$

where $W_{atten}$, $b_{atten}$ and $\nu^{T}$ are learnable parameters. The attention weight $\alpha^{m}$ for each omics is obtained by normalizing the scores across all M=2 omics using softmax:

(8) $$\alpha^{m}=\frac{\exp(c^{m})}{\sum_{j=1}^{M}\exp\left(c^{j}\right)}$$

Finally, the fused intra‐spot multi‐omics representation is computed as a weighted combination:

(9) $$s=\sum_{m=1}^{M}\alpha^{m}\cdot p^{m}$$

yielding a comprehensive representation $s\in R^{D}$ that emphasizes the most informative omics in a data‐adaptive manner.

### 4.3 Inter‐Spot Graph Modeling

#### 4.3.1 Construction of Node Graphs

##### 4.3.1.1 Spatial Proximity Graph

The spatial proximity graph, denoted $G^{s}=\left(V,E^{s}\right)$, is constructed over the set V of N spots. Its adjacency matrix $A^{s}\in R^{N\times N}$ is defined such that $A^{s}\left(i,j\right)=1$ if the Euclidean distance between i‐th spot and j‐th spot is less than a predefined radius threshold r, otherwise 0.

##### 4.3.1.2 Feature Proximity Graph

The feature proximity graph $G^{f}=\left(V,E^{f}\right)$ shares the same node set V and is derived using weighted nearest neighbor (WNN) analysis  [12], which captures multimodal similarity between spots. The adjacency matrix $A^{f}\in R^{N\times N}$ satisfies $A^{f}\left(i,j\right)=1$ if j‐th spot is among the WNN neighbors of i‐th spot, and 0 otherwise.

#### 4.3.2 Chaining Dual Contexts with Graph Attention Network

We employed a two‐layer Graph Attention Network (GAT) to encode the dual‐context graphs, specifically the spatial proximity graph $G^{s}$ and the feature proximity graph $G^{f}$. Unlike the intra‐spot molecular graphs that relied on reliable biological priors, the inter‐spot graphs were purely data‐driven and inherently noisy due to the heterogeneous tissue microenvironment. Physical adjacency in a tissue slice did not strictly guarantee biological similarity, particularly at the boundaries of distinct anatomical structures. If a standard GCN were applied in this context, it would uniformly aggregate features from all adjacent neighbors. This would inevitably lead to over‐smoothing and the blurring of critical structural boundaries. A GAT architecture was specifically chosen to address this challenge. By incorporating a learnable attention mechanism, the model dynamically assigned varying weights to neighbors based on their actual feature representations. This allowed the network to selectively attend to structurally and functionally relevant spots while ignoring irrelevant noise from biologically distinct neighbors, thereby ensuring that fine‐grained spatial domains were sharply delineated.

In our implementation, the first GAT layer aggregated information along feature‐based neighbors, while the second layer aggregated over spatially adjacent neighbors. Formally, let $S\in R^{N\times D}$ denote the multi‐omics representation matrix of all spots, with $s_{i}\in R^{D}$ representing the i‐th spot. The spatial and feature neighbor sets of the i‐th spot are denoted as $S_{i}$ and $F_{i}$, respectively. In each GAT layer, the updated representation for i‐th spot is computed as:

(10) $${\tilde{s}}_{i}=\sigma \left(\sum_{j\in N_{i}}\beta_{ij}\left(W^{l-1}s_{j}+b^{l-1}\right)\right)$$

where the attention weight $\beta_{ij}$ between spots i and j is given by:

(11) $$\beta_{ij}=\frac{\exp\left(\mathrm{LeakyReLU}\left(a^{T}\left[\left(W^{l-1}s_{i}+b^{l-1}\right)⊕\left(W^{l-1}s_{j}+b^{l-1}\right)\right]\right)\right)}{\sum_{k\in N_{i}}\exp\left(\mathrm{LeakyReLU}\left(a^{T}\left[\left(W^{l-1}s_{i}+b^{l-1}\right)⊕\left(W^{l-1}s_{k}+b^{l-1}\right)\right]\right)\right)}$$

here, W and b are learnable parameters, ⊕ denotes vector concatenation, and σ(·) is a nonlinear activation. In the first layer, $N_{i}=S_{i}$; in the second, $N_{i}=F_{i}$. The final output $\tilde{S}\in R^{N\times M}$ represents the context‐aware multi‐omics embeddings.

#### 4.3.3 Self‐Supervised Graph Contrastive Learning for Top‐Down Learning

We employed a self‐supervised contrastive learning (CL) framework to train SMOReg by leveraging both spatial and feature‐based contexts. For each i‐th spot, we aggregated the hidden embeddings of its spatial neighbors and feature‐similar neighbors to form a positive representation $a_{i}$. This design incorporated not only spatially adjacent spots but also functionally similar yet spatially distant ones, enhancing the reliability of positive samples.

To construct negative samples, we shuffled all node features across the dual‐context graph while preserving the original graph structure. The shuffled features $S^{\prime }\in R^{N\times D}$ are then processed by the same GAT encoder as in Equation (12), yielding negative embeddings ${\tilde{S}}^{\prime }\in R^{N\times M}$. For i‐th spot, the tuple $\left({\tilde{s}}_{i},a_{i}\right)$ constitutes a positive pair, while $\left({\tilde{s}}_{i}^{\prime },a_{i}\right)$ forms a negative pair. The objective was to maximize mutual information for positive pairs and minimize it for negative ones, which did not reflect meaningful spatial or functional relationships.

The contrastive loss is defined using binary cross‐entropy (BCE):

(12) $$L_{\text{CL}}=-\frac{1}{2N}\sum_{i=1}^{N}\left(E\left[\log\phi \left({\tilde{s}}_{i},a_{i}\right)\right]+E\left[\log\left(1-\phi \left({\tilde{s}}_{i}^{\prime },a_{i}\right)\right)\right]\right)$$

where $\phi \left(\cdot \right):R^{d}\times R^{d}\to R$ is a discriminator implemented as a two‐layer neural network that estimates the likelihood of a pair being positive. Here, $\phi \left({\tilde{s}}_{i},a_{i}\right)$ denotes the probability score assigned to the positive pair $\left({\tilde{s}}_{i},a_{i}\right)$. Specifically, the discriminator D is implemented as a two‐layer multi‐layer perceptron (MLP). It takes the concatenated vector of the local spot embedding and the global summary graph representation as input. To ensure full algorithmic reproducibility, we specify its precise architectural settings as follows. The first linear layer maps the concatenated input to a hidden space of dimension D (where D equals the dimension of the learned node embeddings), followed by a Parametric Rectified Linear Unit (PReLU) activation function to introduce non‐linearity. The second linear layer projects this hidden representation down to a single scalar value. Finally, a Sigmoid activation function was applied to this scalar to output the final probability score $\phi \left({\tilde{s}}_{i},a_{i}\right)\in \left(0,1\right)$, effectively quantifying the agreement between the local and global representations.

To optimize the SMOReg framework, the model was trained for a fixed duration of 100 epochs across all experiments. Based on our preliminary empirical evaluations, we observed that the contrastive loss consistently reaches stable convergence within this timeframe for all evaluated spatial multi‐omics datasets. Consequently, this fixed‐epoch strategy was uniformly adopted to ensure a standardized experimental setup and to demonstrate the robust convergence properties of the model, rendering additional early stopping mechanisms unnecessary.

### 4.4 Extraction of Domain‐Specific Gene–Protein Regulatory Pairs

To distill regulatory relationships unique to each spatial domain, we developed an analytical pipeline that refines spot‐level affinity matrices through cluster‐wide statistical validation. Initially, a representative matrix for each domain was constructed by averaging the affinity scores across all constituent spots, thereby highlighting consistent molecular patterns. Candidate interactions were then prioritized based on their absolute affinity values within this aggregate matrix. To ensure spatial specificity, each candidate pair was subjected to a permutation test [52]. The observed affinity within a target cluster was evaluated against a null distribution, generated by iteratively shuffling values across different spatial domains. Interactions reaching statistical significance (p<0.05) were categorized as domain‐distinctive, as their regulatory strength significantly exceeded the background levels found in other regions. By mapping these significant pairs to their respective gene and protein identifiers, we produced a refined catalog of salient interactions that characterize the molecular landscape of each tissue compartment.

### 4.5 Functional Characterization of Regulatory Relationships via DAVID Annotation

To contextualize the inferred gene–protein interactions, we conducted functional enrichment analysis using the Database for Annotation, Visualization and Integrated Discovery (DAVID) [53, 54]. After model training, high‐weight interactions were extracted from the spot‐specific affinity matrices generated by SMOReg for downstream evaluation. The associated gene identifiers were subsequently submitted to DAVID's Functional Annotation module (v2024q4), which consolidates up‐to‐date biological knowledge from resources such as NCBI [55], UniProt [56], and various pathway databases including KEGG [57], Reactome [58], and WikiPathways [59]. Enrichment was assessed across a diverse range of knowledge categories, spanning Gene Ontology (GO) terms, protein–protein interaction networks (e.g., BIOGRID, INTACT, MINT), and established pathway resources. By integrating these multifaceted annotations, we were able to map latent regulatory patterns to specific biological processes, thereby facilitating a more comprehensive understanding of the domain‐specific molecular machinery. A gene–protein regulatory pair was considered biologically supported if both molecules co‐occurred in the same significantly enriched pathway or interaction network. These validated relationships were then interpreted within their spatial tissue context, linking domain‐specific regulatory mechanisms to localized biological processes.

## Conflicts of Interest

The authors declare no conflicts of interest.

## Supporting information


**Supporting File**: advs75574‐sup‐0001‐SuppMat.pdf.

## Data Availability

The spatial multi‐omics datasets used in this study are publicly available from previous publications and repositories. The human lymph node dataset, generated by Long et al. [18], using the Visium CytAssist platform, is available at the GEO (accession no. GSE263617) at https://www.ncbi.nlm.nih.gov/geo/query/acc.cgi?acc=GSE263617. The human tonsil dataset, published by Liu et al. [11] and profiled using spatial‐CITE‐seq, can be accessed via GEO (accession no. GSE213264) at https://www.ncbi.nlm.nih.gov/geo/query/acc.cgi?acc=GSE213264. The high‐resolution microscope image of a human tonsil sample is available at https://doi.org/10.6084/m9.figshare.20723680. The mouse thymus dataset, profiled using the Stereo‐CITE‐seq technology, is publicly accessible and can be downloaded directly from the STOmics open‐data portal at https://www.stomics.tech/col1177.
