# Supplementary material for: Decoding Spatial Heterogeneity and Multi‐Omics Regulation with Hierarchical Graph Learning
Source: Adv Sci (Weinh). 2026 May 10;13(43):e75574. doi: 10.1002/advs.75574 (PMC13336022; doi:10.1002/advs.75574)
Supplement: Supplementary file 1 — Supporting File: advs75574‐sup‐0001‐SuppMat.pdf. [file ADVS-13-e75574-s001.pdf]

Supplementary materials

Jiazhou Chen[1], Jiahui Xie[2], Yi Liao[2], Junyu Li[3], Longqi Liu[4], Xin Gao[5,6,7], Hongmin Cai[3,*]

[1]School of Computer Science and Technology, Guangdong University of Technology, Guangzhou, Guangdong, China
[2]School of Computer Science and Technology, South China University of Technology, Guangzhou, Guangdong, China
[3]School of Future Technology, South China University of Technology, Guangzhou, Guangdong, China
[4]BGI-Shenzhen, 518103 Shenzhen, China
[5]Computer Science Program, Computer, Electrical and Mathematical Sciences and Engineering Division, King Abdullah University of Science and Technology (KAUST), King Abdullah, Kingdom of Saudi Arabia
[6]Center of Excellence for Smart Health (KCSH), King Abdullah University of Science and Technology, King Abdullah, Kingdom of Saudi Arabia
[7]Center of Excellence on Generative AI, King Abdullah University of Science and Technology, King Abdullah, Kingdom of Saudi Arabia

Supplementary Notes

Supplementary Note 1: Generation of simulated multi-Omics spatial data with various noise levels

We generated synthetic multi-omics datasets by introducing Gaussian noise at multiple levels to a shared baseline simulation. Transcriptomic profiles were simulated using a zero-inflated negative binomial model, with 50% of values set to zero to mimic dropout effects, while proteomic data were drawn from a standard negative binomial distribution reflecting their typically higher detection sensitivity. Each dataset contained 2,500 spatial spots. The transcriptome included 3,000 genes (Supplementary Fig. 1a) and the proteome 200 proteins (Supplementary Fig. 1b). We defined four spatial patterns and one background region. Spots 0–336 were assigned to pattern 1, 337–752 to pattern 2, 753–1207 to pattern 3, and 1208–1576 to pattern 4; the rest formed the background (Fig. 2a). Genes and proteins were evenly divided into four groups, each uniquely overexpressed in one spatial pattern. Regulatory relationships were embedded by linking overexpression regions across omics—e.g., transcriptomic columns 0–749 and proteomic columns 0–49 were co-regulated in pattern 1. Finally, we created eight noise levels by adding zero-mean Gaussian noise with variance from 50% to 200% of the mean expression, simulating increasing measurement distortion.

Supplementary Note 2: Downstream task analysis scheme

For each spatial domain, we aggregated the corresponding regulatory affinity matrices across all assigned spots and extracted high-affinity gene–protein pairs. Functional enrichment analysis was performed using DAVID[1,2] to associate these regulatory pairs with relevant biological processes and pathways. Results were visualized using a Sankey-bubble plot, linking enriched terms to their molecular contributors, with bubble size and color indicating the number of molecules and fold enrichment, respectively. Domain-specific regulatory interactions were simultaneously visualized via chord diagrams, using a shared node set and consistent coloring to enable cross-domain comparison. This analytical scheme was applied uniformly across all experimental datasets.

Supplementary Note 3: Details of domain-specific molecular regulatory mechanisms on the human lymph node

In cluster 3 (capsule and follicular region), the *CXCL13*–CXCR5 regulatory pair is linked to extrafollicular and follicular B cell activation. Follicles, situated beneath the capsule, are rich in B cells and serve as key sites for antigen

*All correspondence should be addressed to Hongmin Cai(hmcai@scut.edu.cn).

encounter and activation[3]. *CXCL13* binding to CXCR5 guides B cell migration and supports germinal center formation[4], a relationship also reflected in the KEGG *Cytokine-cytokine receptor interaction* (Supplementary Fig. 5e).

In cluster 5 (paracortex), the *CCL21*–CCR7 pair is enriched for *chemokine receptor activity in T cells*. CCR7 and *CCL21* form a chemotactic axis that directs T cells and dendritic cells into the T cell zone[5,6], with disrupted signaling impairing immune localization[7]. This pair is also annotated in the KEGG *Chemokine signaling pathway* (Supplementary Fig. 7). Additionally, *CCL21*–CD4 is associated with *positive regulation of ERK1/2 (Extracellular signal-Regulated Kinase 1 and 2) cascade*, where *CCL21* promotes ERK phosphorylation to enhance $CD4^+$ T cell proliferation and polarization[8].

In cluster 6 (pericapsular adipose tissue), the *CD22* molecule—involving *FABP4* (lipid metabolism) and CD19 (BCR coreceptor)—connects lipid-mediated inflammation to immune regulation near follicles. Inflammatory signals such as LPS, commonly present in perinodal adipose tissue, can modulate CD22 to suppress BCR signaling[9].

Supplementary Note 4: Ablation study on the cross-graph matching module

To demonstrate the specific contribution of the cross-graph matching module to the overall clustering performance, we performed an ablation study across three real spatial multi-omics datasets (human lymph node, human tonsil, and mouse thymus). We established a baseline model, denoted as SMOReg_wo_cross_matching, which removes the cross-graph matching and convolution processes. In this baseline, the molecular features refined by the intra-omics Graph Convolutional Networks (GCNs) are fed directly into the bottom-level dual-attention mechanism for multi-omics fusion, leaving all other architectural components and hyperparameters unchanged.

Qualitative Spatial Mapping Evaluation: The visualization of spatial domains (Supplementary Fig. 15) reveals that the absence of the cross-graph matching module leads to a significant loss of critical biological resolution. In the human lymph node dataset (Supplementary Fig. 15a), the ablated model completely failed to distinguish the cortex from the paracortex—a crucial biological subdivision successfully identified by the full model. Instead, it erroneously introduced an artificial subdivision within the central medulla, leading to a loss of key biological findings that are essential for subsequent regulatory analysis. For the human tonsil (Supplementary Fig. 15b) and mouse thymus (Supplementary Fig. 15c) datasets, while the ablated model could roughly separate broader subtypes (e.g., the germinal center light/dark zones and crypt epithelial vs. peripheral blood cells in the tonsil; the general layered structure in the thymus), the clustering quality severely degraded. The ablation introduced isolated noise spots across the tissue sections, blurred the boundaries between subtypes, and confounded hierarchical structures. Notably, in the mouse thymus, the outmost capsular layer was lost, and the intricate structure of the medulla became spatially mixed and indistinguishable. These qualitative comparisons corroborate that the cross-graph matching mechanism is vital for discovering and refining important spatial sub-domains.

Quantitative Performance Evaluation: Quantitatively, the full SMOReg framework consistently outperformed the ablated baseline across all datasets (Supplementary Fig. 16). Whether evaluated by supervised metrics (e.g., ARI, NMI, Completeness) on the annotated lymph node dataset or unsupervised metrics (e.g., Moran's I, Silhouette Index, Calinski-Harabasz Index) on the tonsil and thymus datasets, the inclusion of the cross-graph matching module yielded substantial and significant metric improvements. Furthermore, the boxplots for the lymph node dataset (Supplementary Fig. 16a) indicate that the full model not only achieves higher scores but also exhibits much smaller fluctuation ranges, demonstrating enhanced algorithmic stability.

Taken together, the ablation study provides direct evidence that the cross-graph matching module is an indispensable component of the SMOReg framework. By enabling explicit cross-omics communication, it captures critical inter-omics regulatory signals that naive fusion strategies overlook. This module directly translates into superior quantitative stability and highly accurate, biologically interpretable spatial domain identification, which serves as the foundation for the downstream molecular regulatory analysis.

Supplementary Note 5: Sensitivity analysis of the WNN neighbor selection strategy

To address the sensitivity of the Weighted Nearest Neighbor (WNN) analysis used in constructing the feature proximity graph, we performed a comprehensive hyperparameter evaluation on the real spatial multi-omics datasets. Specifically, we varied the number of nearest neighbors (k) from 1 to 10 and quantitatively monitored the downstream spatial domain identification performance across multiple clustering metrics (e.g., ARI, AMI, Moran's I). As shown in Supplementary Fig. 17, the performance curves exhibit a highly stable plateau when the number of neighbors is restricted

to $k \leq 7$, with a marginal peak in accuracy observed at approximately $k = 5$. Based on this empirical evidence, $k = 5$ was selected as the default parameter for our main experiments.

Interestingly, a slight downward trend in performance metrics was observed as the neighbor count increased further ($k \geq 8$). We attribute this behavior to the intrinsic design principles of the WNN algorithm rather than a limitation of the SMOReg architecture. The WNN algorithm dynamically calculates modality weights to define a multimodal distance metric, inherently prioritizing the most compositionally similar spots. As the specified number of neighbors increases, the algorithm is forced to traverse larger distances in the latent multimodal space, inevitably incorporating spots that are less functionally or biologically similar. The inclusion of these more distant neighbors introduces background noise and dilutes the localized omics signature, slightly degrading the purity of the feature proximity graph.

Overall, this result is acceptable and demonstrates that our strategy of utilizing the WNN algorithm to filter and restrict the neighborhood size is sound. The consistent performance stability within the $k \in [1, 7]$ range provides strong evidence that SMOReg is robust to initial graph-building settings and that the extracted spatial domains are reliable.

References

[1] Brad T Sherman, Ming Hao, Ju Qiu, Xiaoli Jiao, Michael W Baseler, H Clifford Lane, Tomozumi Imamichi, and Weizhong Chang. David: a web server for functional enrichment analysis and functional annotation of gene lists (2021 update). *Nucleic Acids Research*, 50(W1):W216–W221, 2022.

[2] Da Wei Huang, Brad T Sherman, and Richard A Lempicki. Systematic and integrative analysis of large gene lists using david bioinformatics resources. *Nature Protocols*, 4(1):44–57, 2009.

[3] K Mark Ansel, Vu N Ngo, Paul L Hyman, Sanjiv A Luther, Reinhold Förster, Jonathon D Sedgwick, Jeffrey L Browning, Martin Lipp, and Jason G Cyster. A chemokine-driven positive feedback loop organizes lymphoid follicles. *Nature*, 406(6793):309–314, 2000.

[4] Jason G Cyster. Chemokines and cell migration in secondary lymphoid organs. *Science*, 286(5447):2098–2102, 1999.

[5] Reinhold Förster, Ana Clara Davalos-Misslitz, and Antal Rot. Ccr7 and its ligands: balancing immunity and tolerance. *Nature Reviews Immunology*, 8(5):362–371, 2008.

[6] Luiz Henrique Geraldo, Celina Garcia, Yunling Xu, Felipe Saceanu Leser, Izabella Grimaldi, Eduardo Sabino de Camargo Magalhães, Joost Dejaegher, Lien Solie, Cláudia Maria Pereira, Ana Helena Correia, et al. Ccl21-ccr7 signaling promotes microglia/macrophage recruitment and chemotherapy resistance in glioblastoma. *Cellular and Molecular Life Sciences*, 80(7):179, 2023.

[7] Le Han and Lingling Zhang. Ccl21/ccr7 axis as a therapeutic target for autoimmune diseases. *International Immunopharmacology*, 121:110431, 2023.

[8] Kathrin Gollmer, François Asperti-Boursin, Yoshihiko Tanaka, Klaus Okkenhaug, Bart Vanhaesebroeck, Jeffrey R Peterson, Yoshinori Fukui, Emmanuel Donnadieu, and Jens V Stein. Ccl21 mediates cd4+ t-cell costimulation via a dock2/rac-dependent pathway. *Blood, The Journal of the American Society of Hematology*, 114(3):580–588, 2009.

[9] Frédéric Lajaunias, Lars Nitschke, Thomas Moll, Eduardo Martinez-Soria, Isabelle Semac, Yves Chicheportiche, R Michael E Parkhouse, and Shozo Izui. Differentially regulated expression and function of cd22 in activated b-1 and b-2 lymphocytes. *The Journal of Immunology*, 168(12):6078–6083, 2002.

Supplementary Figures

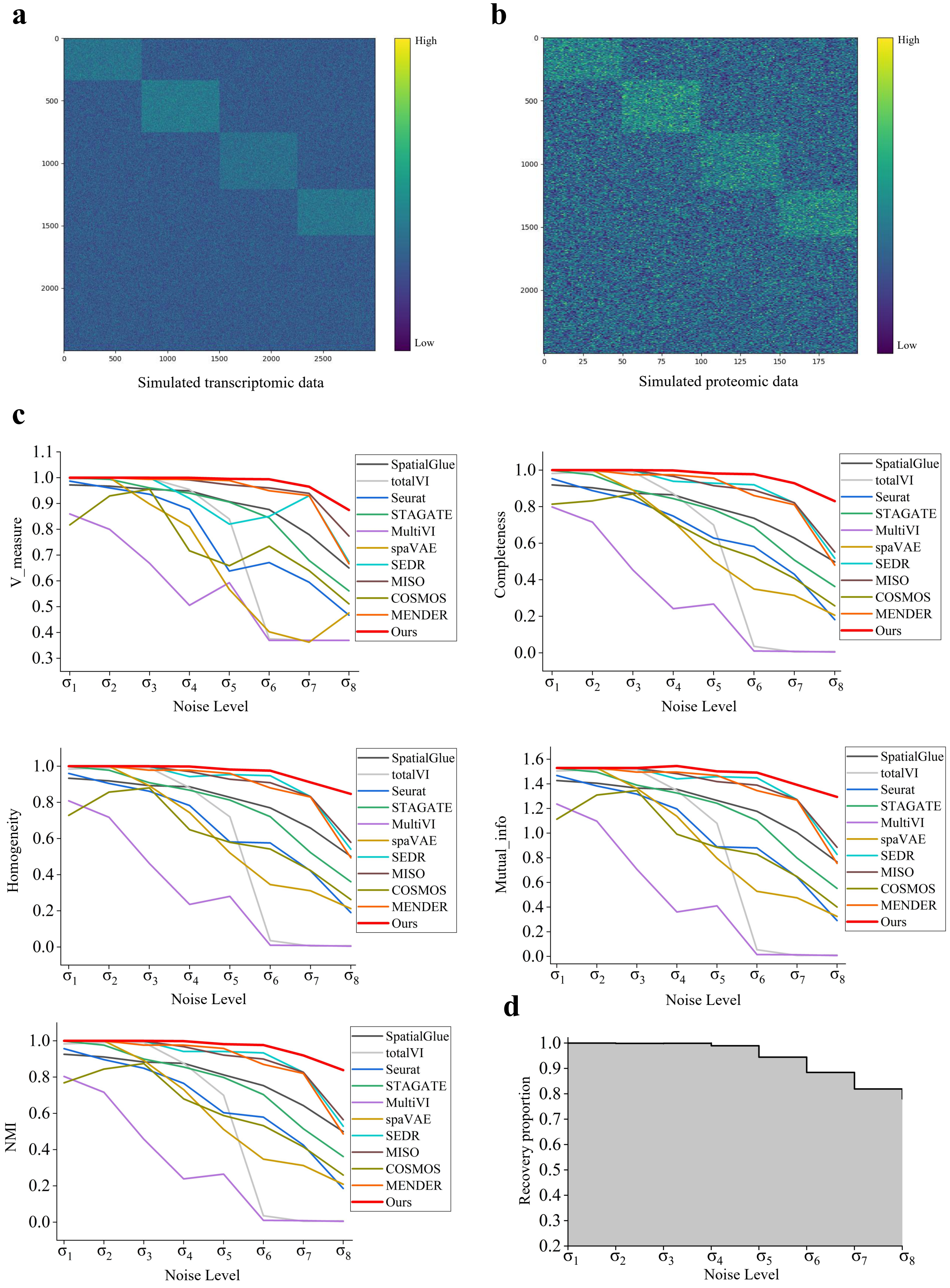

Supplementary Fig. 1: Simulated data visualization and additional benchmarking results. **a**, Heatmap of the simulated transcriptomic matrix (2,500 spots × 3,000 genes). Diagonal blocks correspond to expression patterns 1–4; remaining rows represent background spots. Color intensity reflects expression levels. **b**, Heatmap of the simulated proteomic matrix (2,500 spots × 200 proteins). Diagonal blocks indicate pattern-specific protein features; other rows denote background. Color intensity represents protein abundance. **c**, Performance evaluation of SMOReg and baselines under increasing Gaussian noise ($\sigma 1$–$\sigma 8$), evaluated using V-measure, Completeness, Homogeneity, Mutual Information, and NMI. **d**, Recovery rate of ground-truth regulatory interactions across noise levels. Each noise level applies matched variance to both transcriptomic and proteomic data.

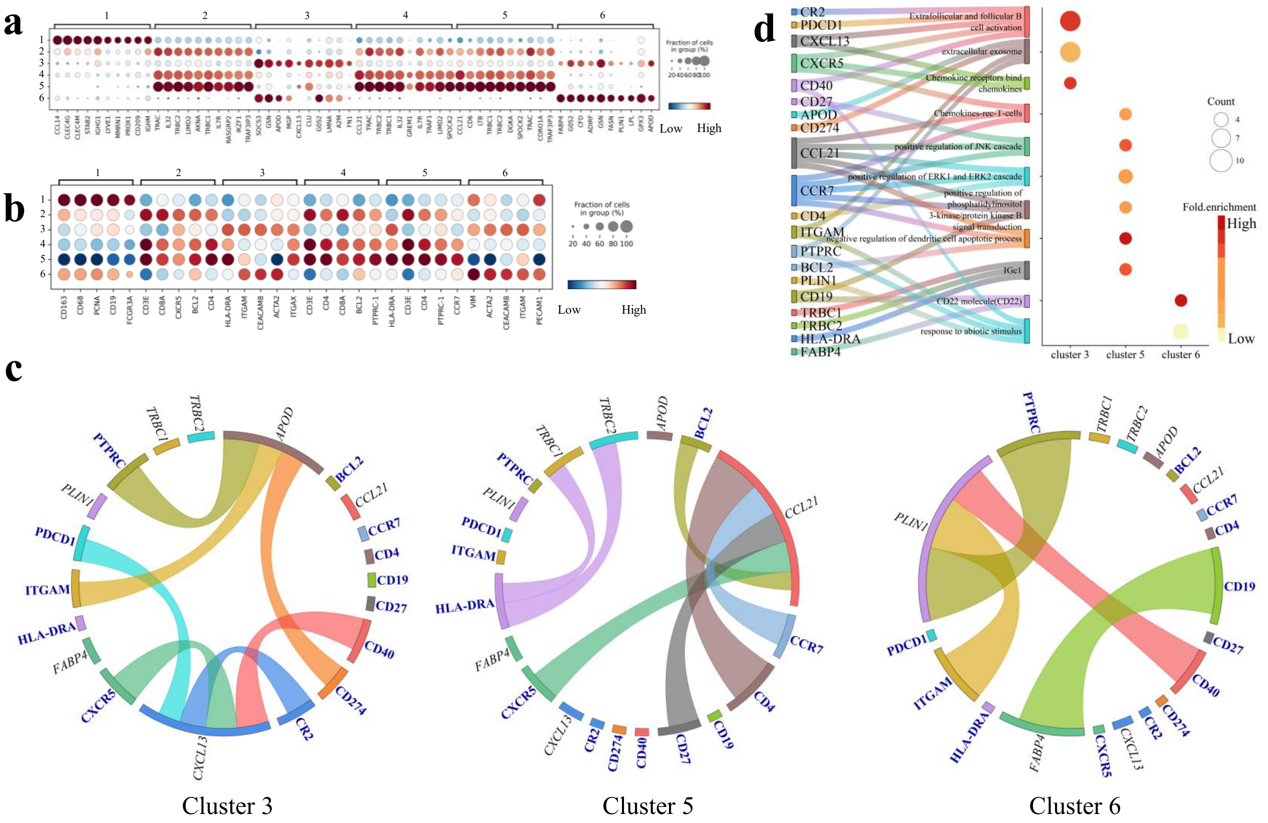

Supplementary Fig. 2: Regulatory analysis of the human lymph node sample. **a**, Transcriptomic differential expression across SMOReg clusters 3, 5, and 6. Dot size represents expressing cell fraction; color indicates the average expression level. **b**, Proteomic differential expression across the same clusters. **c**, Chord diagrams of gene–protein regulatory interactions in SMOReg clusters 3, 5, and 6. Nodes (genes in italics, proteins upright) represent molecules; chords denote high-affinity pairs. All diagrams share a unified node set and consistent coloring. **d**, Sankey-bubble plot linking enriched biological processes to regulatory molecules. Left: molecular contributors; right: enriched terms with bubble color/size showing fold enrichment and molecule count.

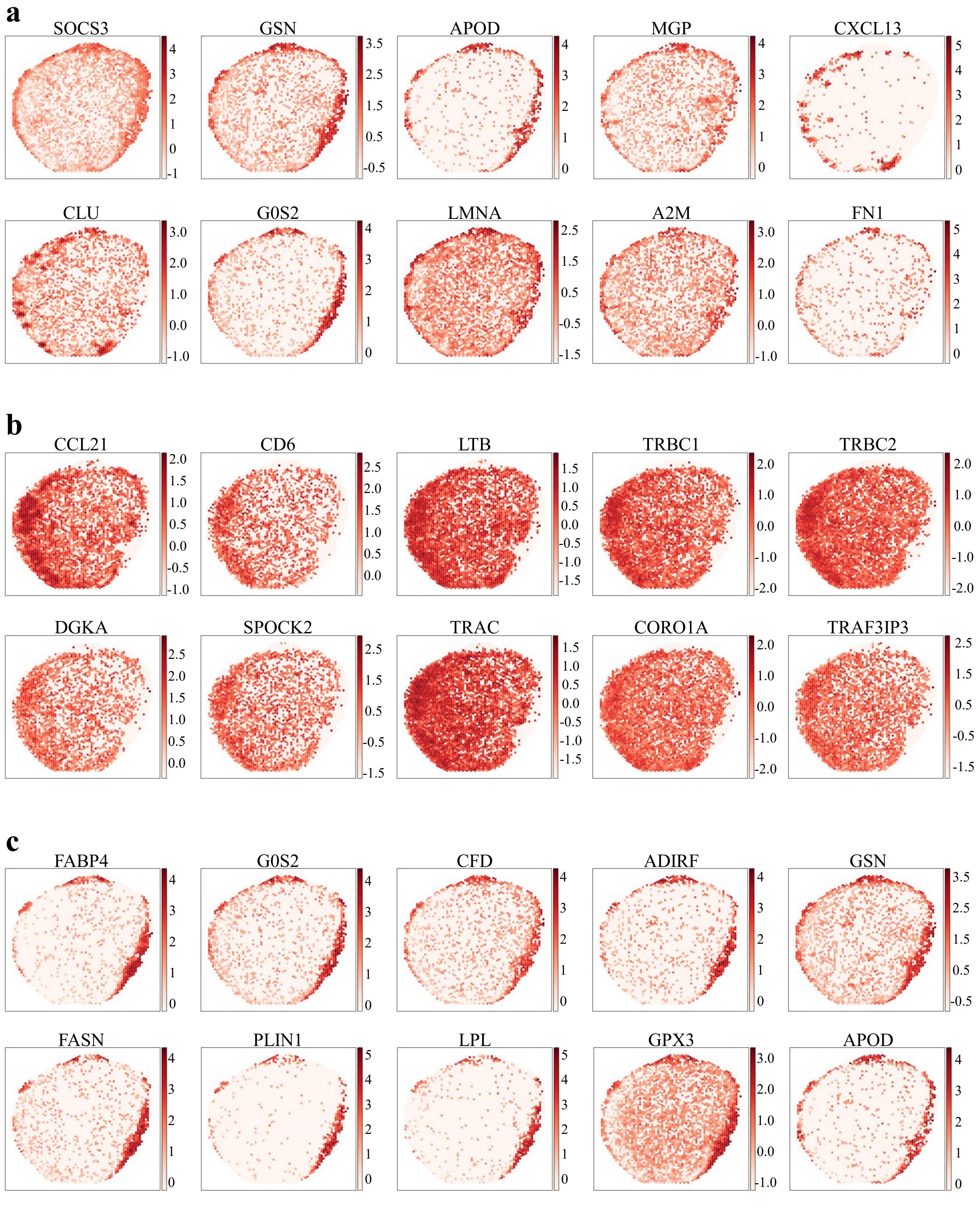

Supplementary Fig. 3: Spatial expression heatmaps of representative genes differentially expressed in three spatial domains of the human lymph node. **a**, SMOReg cluster 3 (the capsular zone). **b**, SMOReg cluster 5 (the paracortex domain). **c**, SMOReg cluster 6 (the pericapsular adipose tissue). The color intensity reflects the relative expression level of each molecule, quantified as z-scores after data scaling, with darker shades indicating higher standardized values.

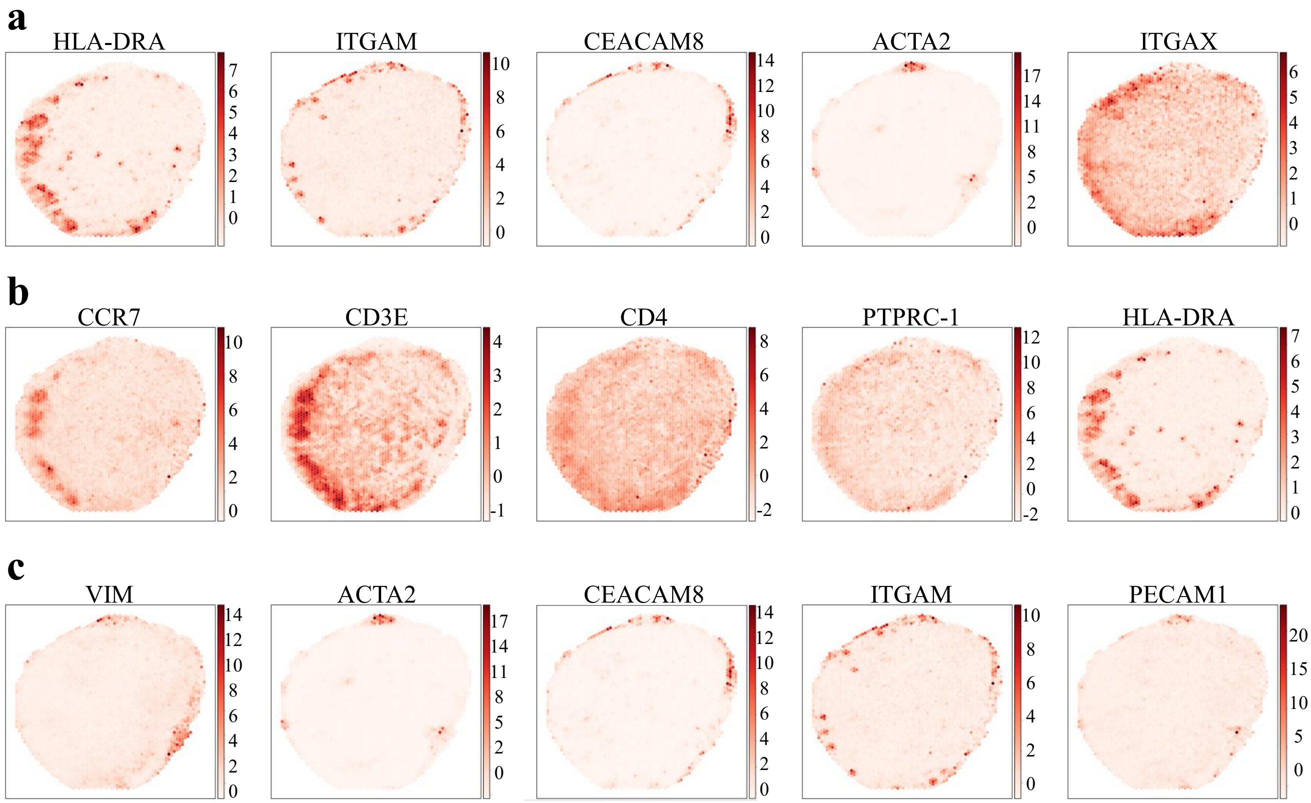

Supplementary Fig. 4: Spatial expression heatmaps of representative proteins differentially expressed in three spatial domains of the human lymph node sample. **a**, SMOReg cluster 3 (the capsular zone). **b**, SMOReg cluster 5 (the paracortex domain). **c**, SMOReg cluster 6 (the pericapsular adipose tissue). The color intensity reflects the relative abundance level of each molecule, quantified as z-scores after data scaling, with darker shades indicating higher standardized values.

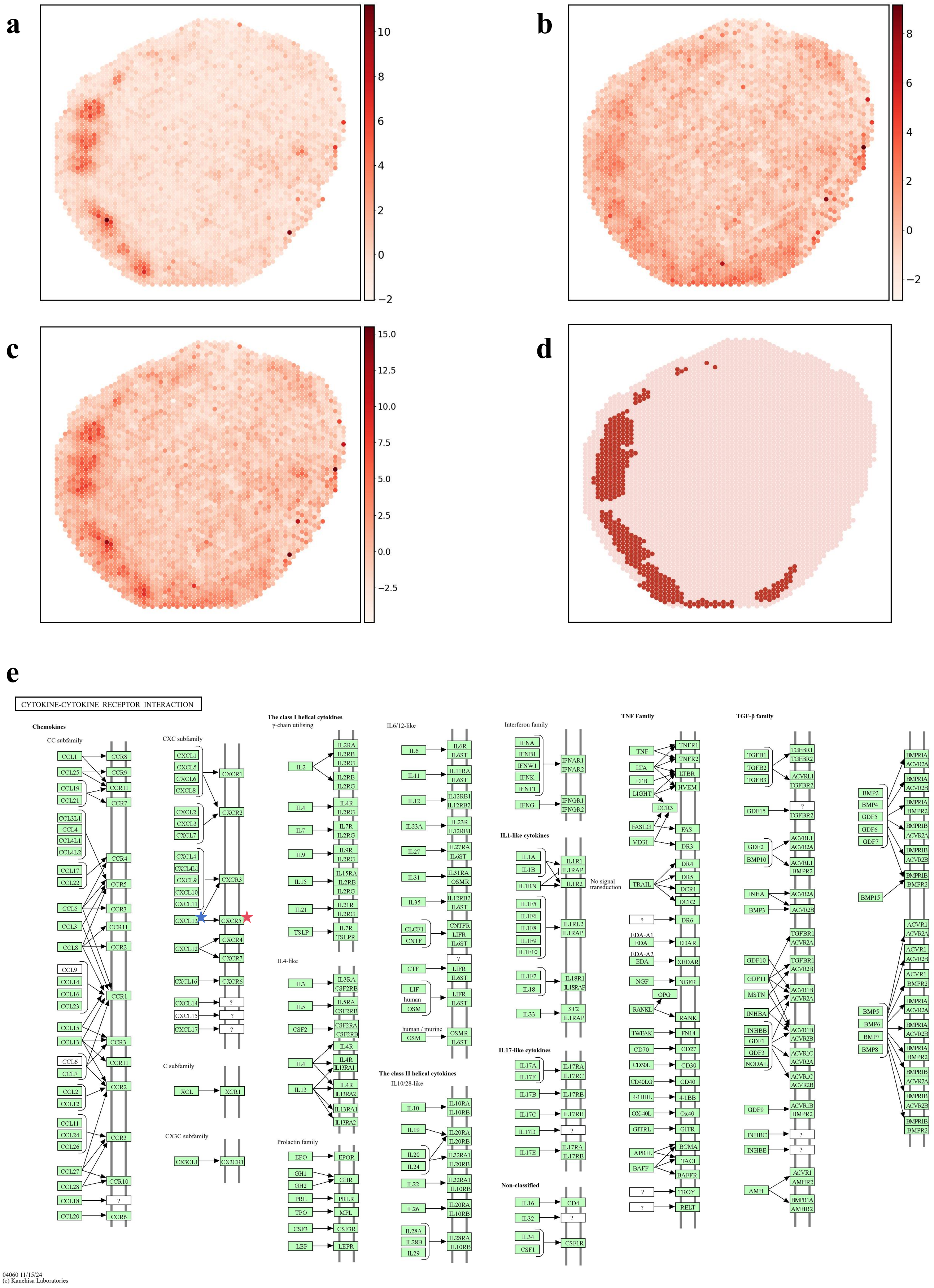
a
b
c
d
e
CYTOKINE-CYTOKINE RECEPTOR INTERACTION
Chemokines
CC subfamily
CXC subfamily
C subfamily
CX3C subfamily
The class I helical cytokines
γ-chain utilising
IL4-like
Prolactin family
IL6/12-like
The class II helical cytokines
IL10/28-like
Interferon family
IL1-like cytokines
No signal transduction
IL17-like cytokines
Non-classified
TNF Family
TGF-β family
04060 11/15/24
(c) Kanehisa Laboratories

Supplementary Fig. 5: Additional results for human lymph node analysis. a–c, Spatial expression patterns of CCR7 (a), CD4 (b), and their overlay (c) in the human lymph node section. Color intensity reflects protein abundance, with darker colors indicating higher expression. **d**, Spatial distribution of SMOReg cluster 5, corresponding to the paracortex region. **e**, KEGG pathway map (hsa04060) of cytokine–cytokine receptor interactions. The highlighted CXCL13–CXCR5 interaction is marked in blue and red, respectively, within the chemokine signaling network.

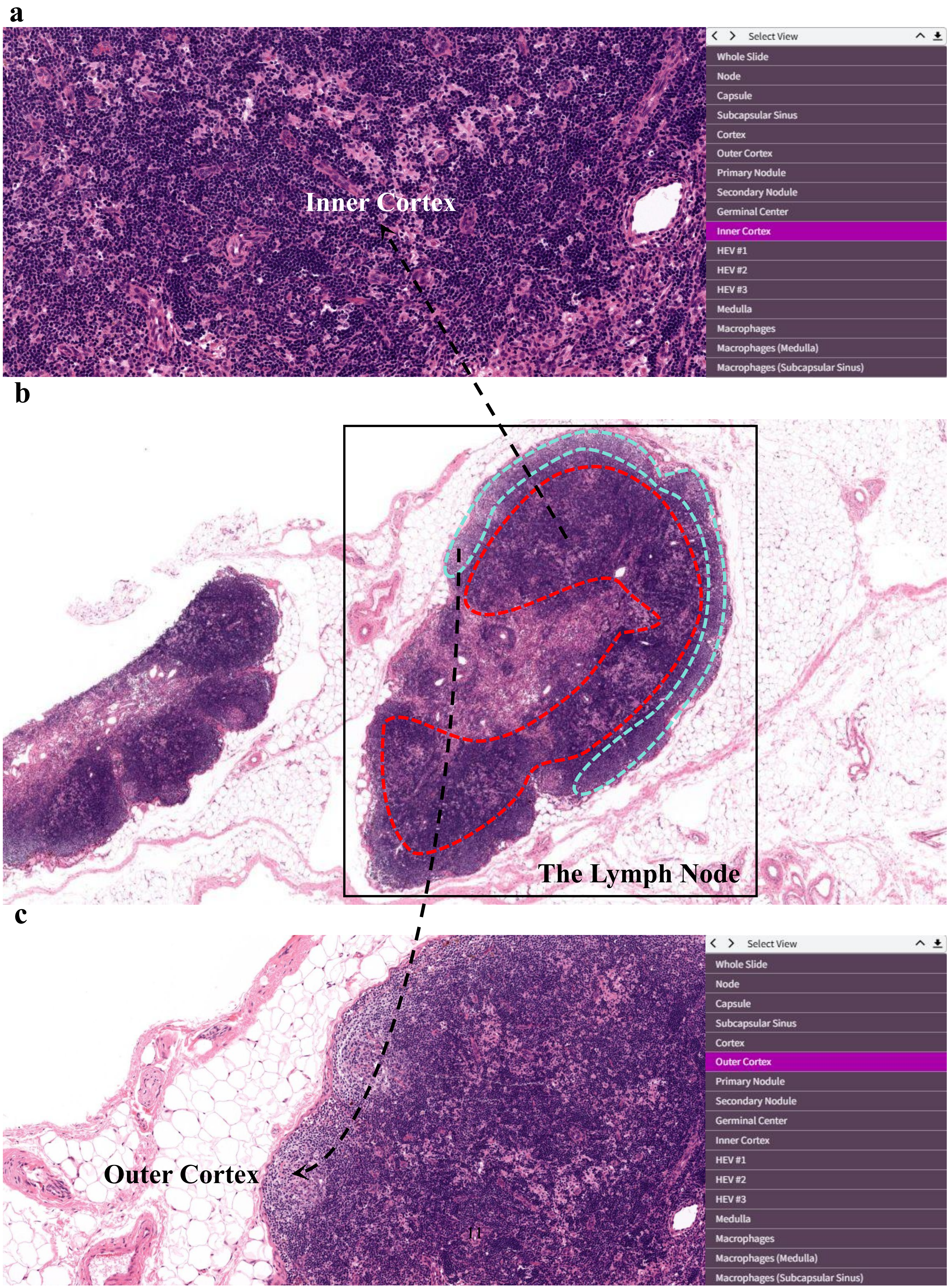
a
Select View
Whole Slide
Node
Capsule
Subcapsular Sinus
Cortex
Outer Cortex
Primary Nodule
Secondary Nodule
Germinal Center
Inner Cortex
HEV #1
HEV #2
HEV #3
Medulla
Macrophages
Macrophages (Medulla)
Macrophages (Subcapsular Sinus)
Inner Cortex
b
The Lymph Node
c
Select View
Whole Slide
Node
Capsule
Subcapsular Sinus
Cortex
Outer Cortex
Primary Nodule
Secondary Nodule
Germinal Center
Inner Cortex
HEV #1
HEV #2
HEV #3
Medulla
Macrophages
Macrophages (Medulla)
Macrophages (Subcapsular Sinus)
Outer Cortex

Supplementary Fig. 6: Histological images of human lymph node (H&E stain, MHS 213 Lymph Nodes, Histology Guide). **a**, High-magnification view of the inner cortex (paracortex), selected via the “Inner Cortex” dropdown annotation. The region appears as a uniform, densely packed deep purple area. **b**, Overview of the full lymph node section. A black solid rectangle encloses the entire lymph node. The inner cortex (paracortex) is outlined by a red dashed line, and the outer cortex by a cyan dashed line. Black dashed arrows point to the magnified regions shown in **a** and **c**. **c**, High-magnification view of the outer cortex, selected via the “Outer Cortex” dropdown annotation. The region displays a pale purple outer layer surrounding darker areas.

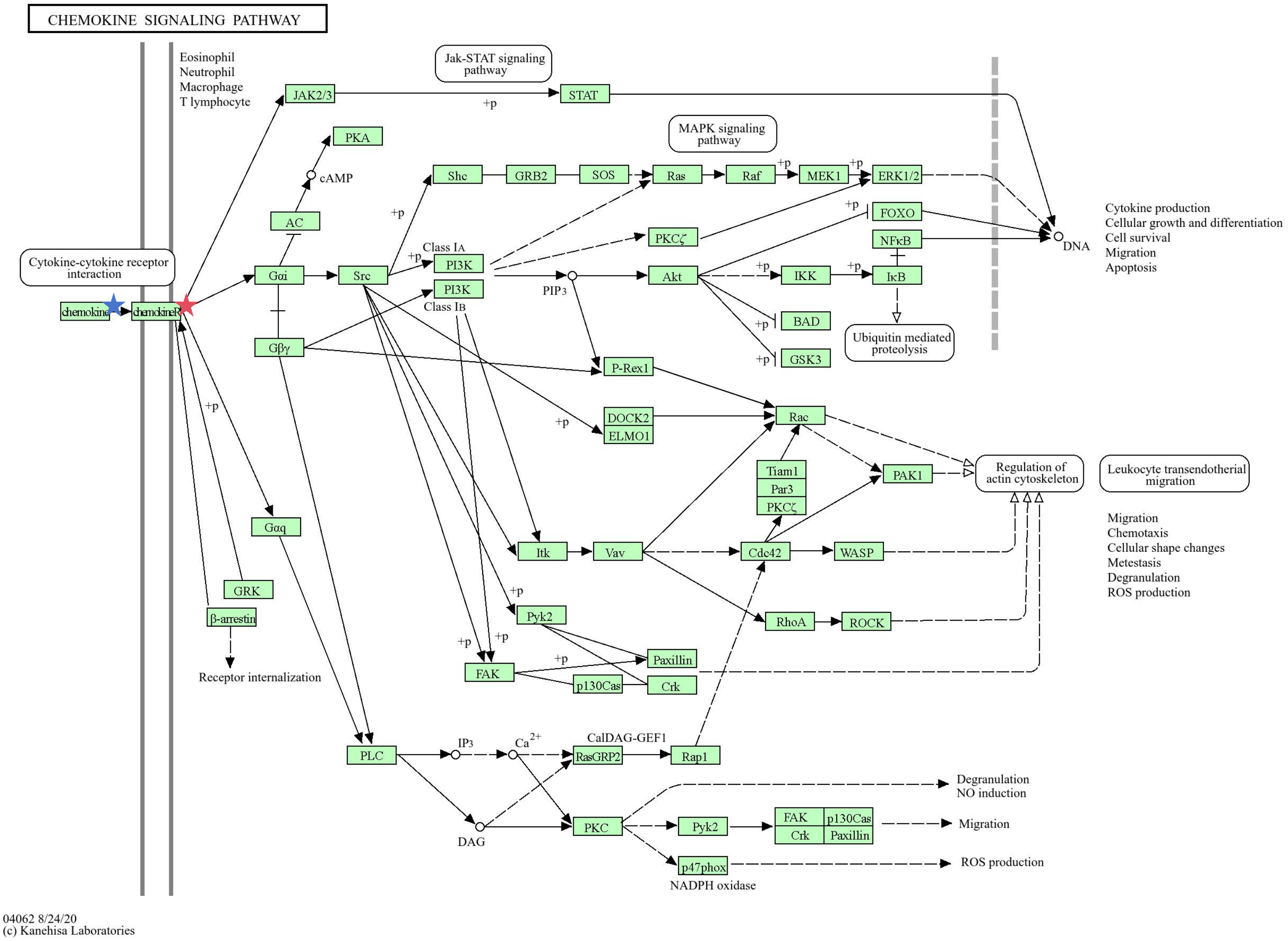

Supplementary Fig. 7: KEGG pathway map (hsa04062) of the Chemokine signaling pathway. This schematic illustrates downstream signaling events initiated by chemokine–receptor binding in immune cells. The blue star marks a chemokine entry that includes CCL21, while the red star highlights its receptor CCR7. The presence of both indicates a functional ligand–receptor interaction, relevant to the spatial regulatory mechanisms discussed in this study.

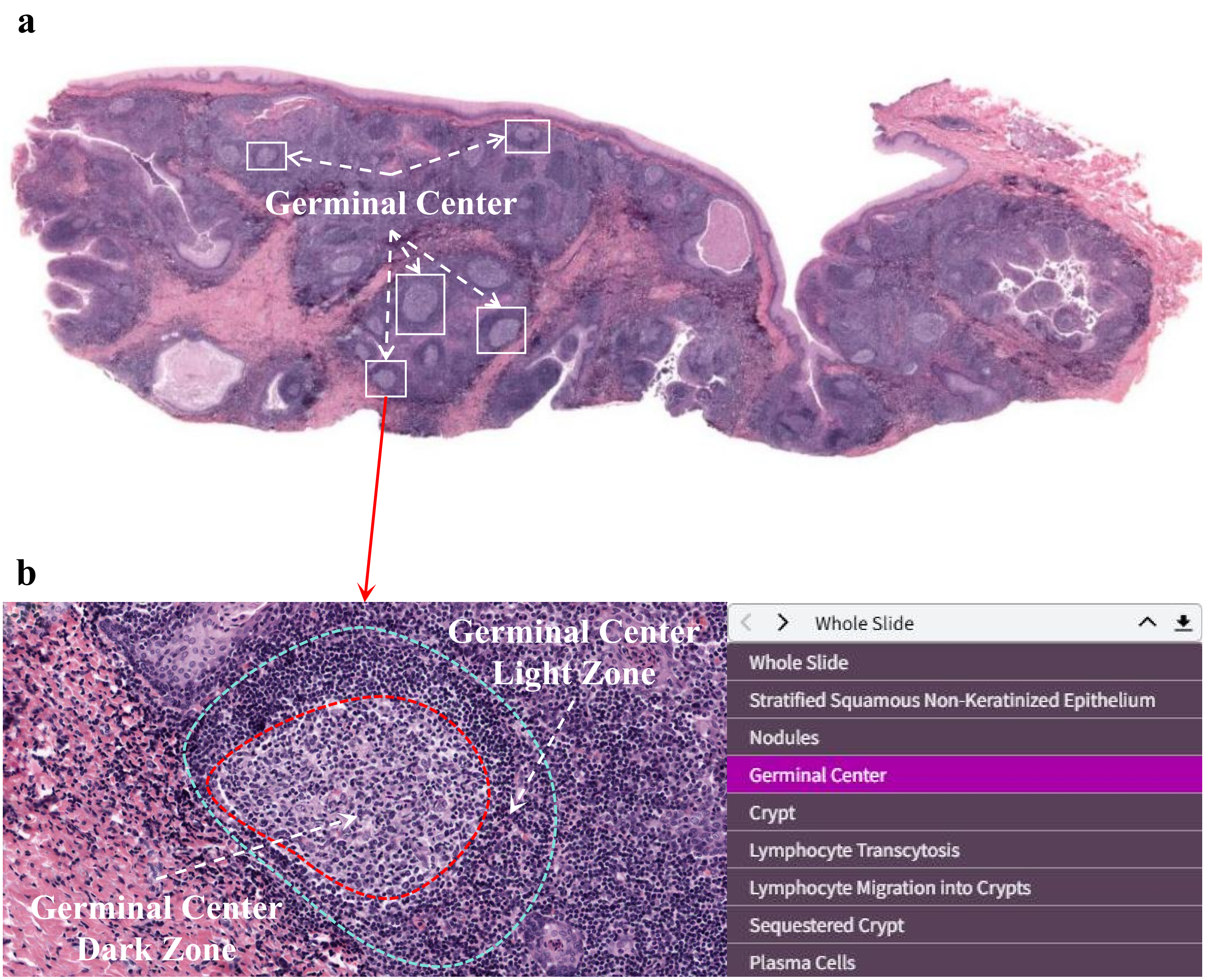

Supplementary Fig. 8: Histological evidence of germinal center light and dark zones in human tonsil (H&E stain, MH 081a Palatine Tonsil, Histology Guide). **a**, Overview of an H&E-stained section of human tonsil. White rectangles highlight several germinal centers within the lymphoid tissue. **b**, High-magnification view of one germinal center selected from **a**. The light zone is outlined by a cyan dashed line and shows densely packed cells; the dark zone is enclosed by a red dashed line and displays relatively sparse cellular arrangement.

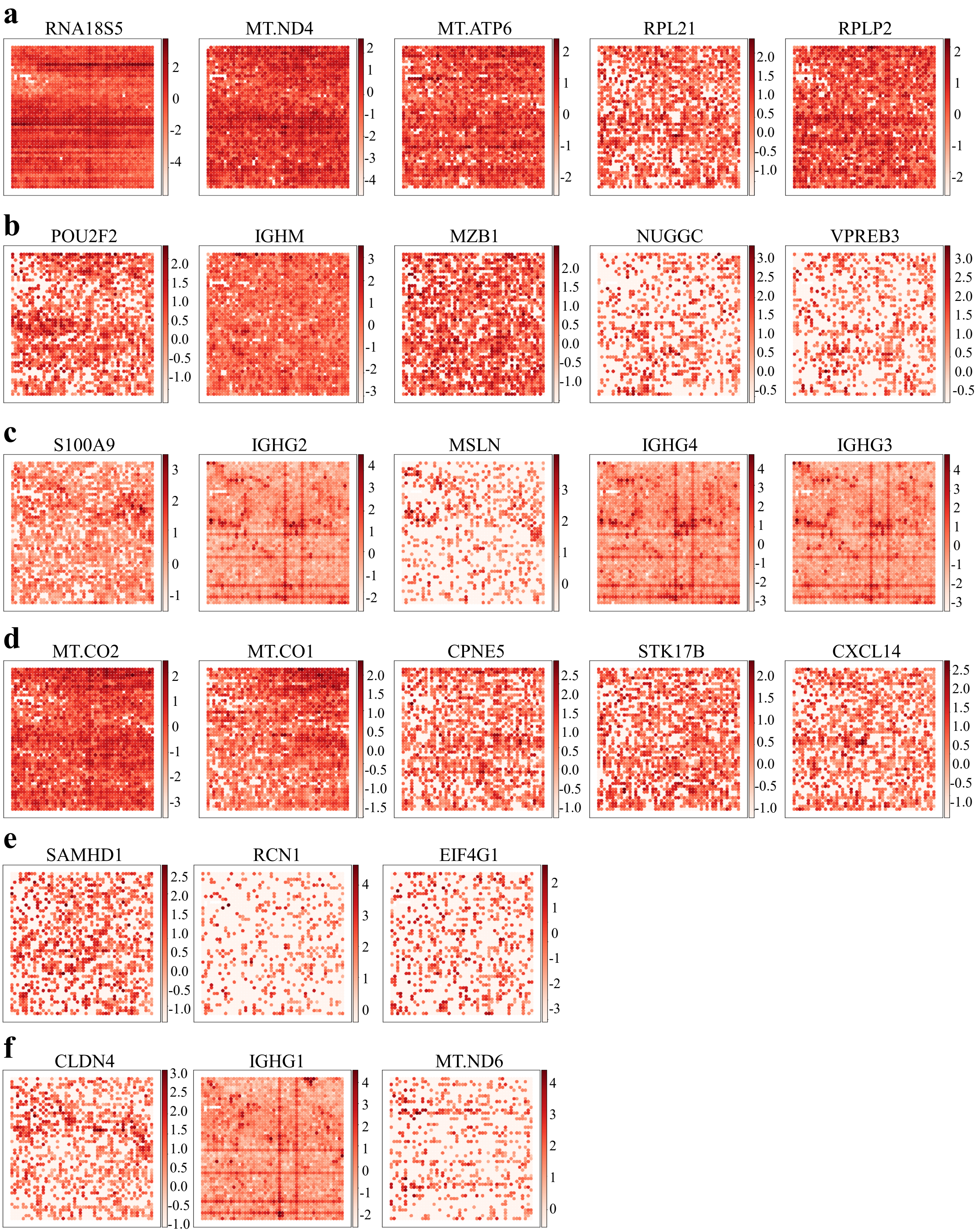

Supplementary Fig. 9: Spatial expression heatmaps of representative genes differentially expressed in each spatial domain of the human tonsil sample. **a**, SMOReg cluster 1 (the T cell zone). **b**, SMOReg cluster 2 (the germinal center dark zone). **c**, SMOReg cluster 3 (the crypt epithelial region). **d**, SMOReg cluster 4 (the germinal center light zone). **e**, SMOReg cluster 5 (the extrafollicular region). **f**, SMOReg cluster 6 (the peripheral blood cell region). The color intensity reflects the relative expression level of each molecule, quantified as z-scores after data scaling, with darker shades indicating higher standardized values.

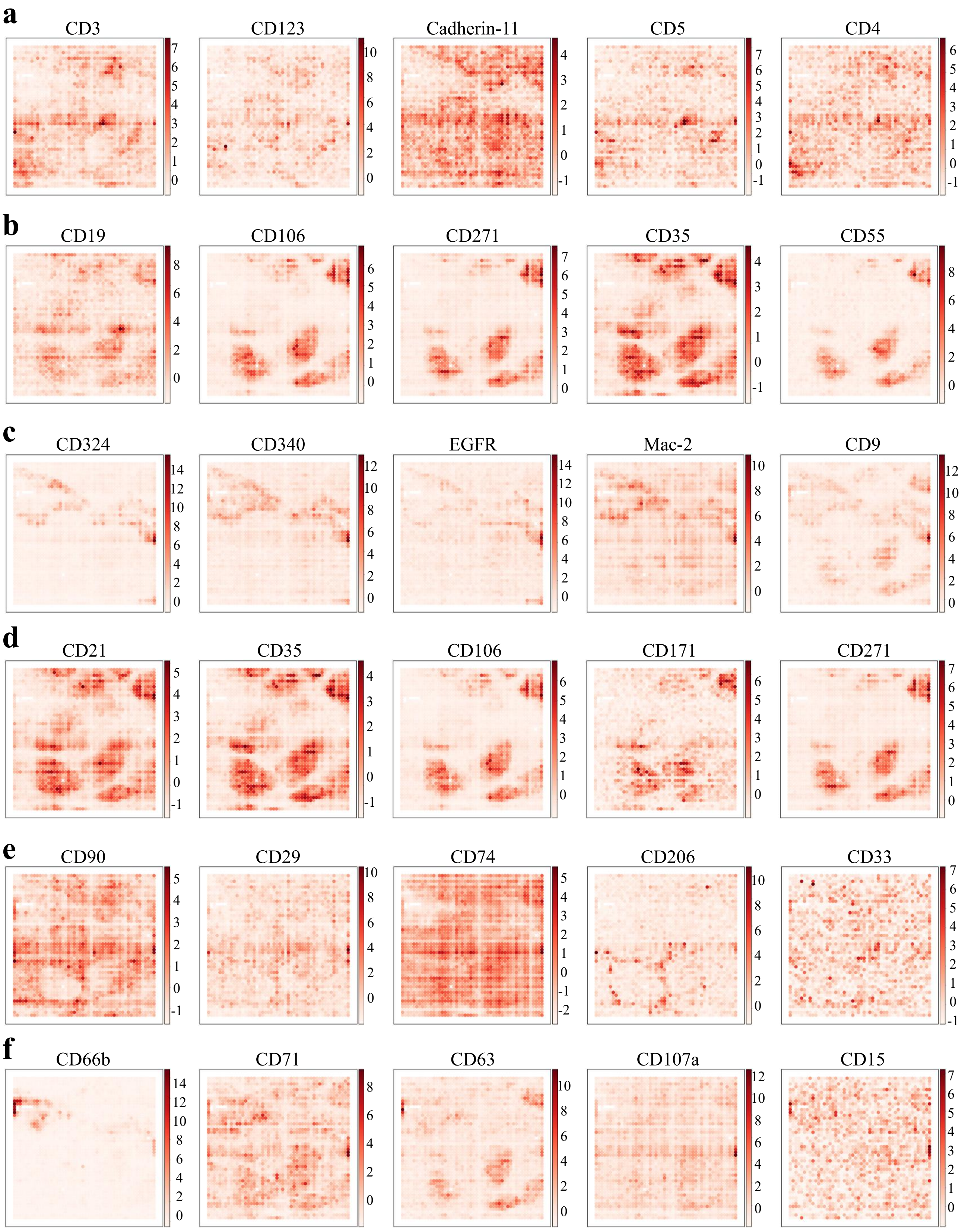

Supplementary Fig. 10: Spatial expression heatmaps of representative proteins differentially expressed in each spatial domain of the human tonsil sample. **a**, SMOReg cluster 1 (the T cell zone). **b**, SMOReg cluster 2 (the germinal center dark zone). **c**, SMOReg cluster 3 (the crypt epithelial region). **d**, SMOReg cluster 4 (the germinal center light zone). **e**, SMOReg cluster 5 (the extrafollicular region). **f**, SMOReg cluster 6 (the peripheral blood cell region). The color intensity reflects the relative abundance level of each molecule, quantified as z-scores after data scaling, with darker shades indicating higher standardized values.

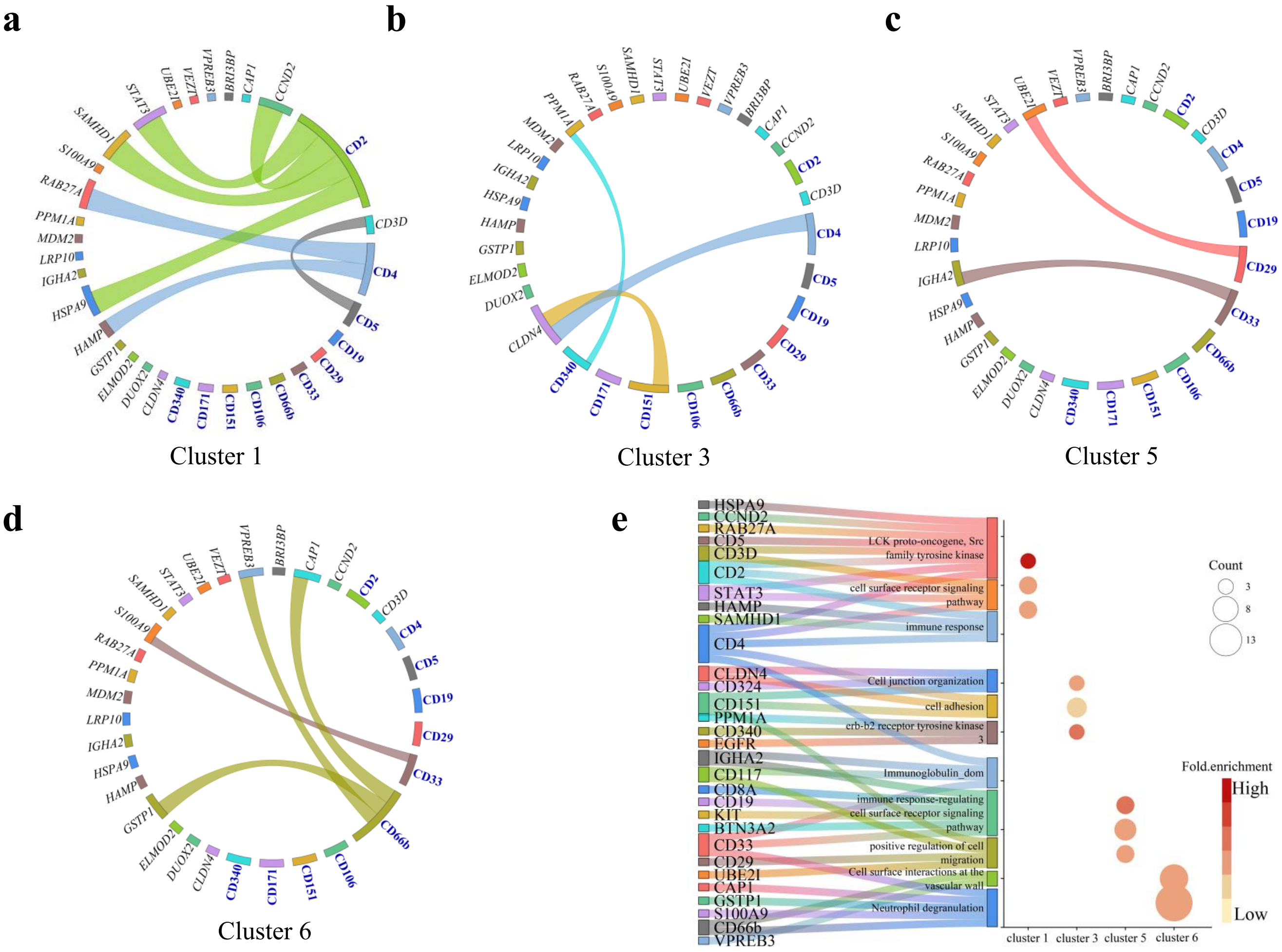

Supplementary Fig. 11: Additional regulatory analysis of the human tonsil sample. **a**, Chord diagrams of regulatory interactions of SMOReg cluster 1. **b**, Chord diagrams of regulatory interactions of SMOReg cluster 3. **c**, Chord diagrams of regulatory interactions of SMOReg cluster 5. **d**, Chord diagrams of regulatory interactions of SMOReg cluster 6. **e**, Sankey-bubble plot linking enriched biological processes to regulatory molecules for the remaining SMOReg clusters. Left: molecular contributors; right: enriched terms with bubble color/size showing fold enrichment and molecule count.

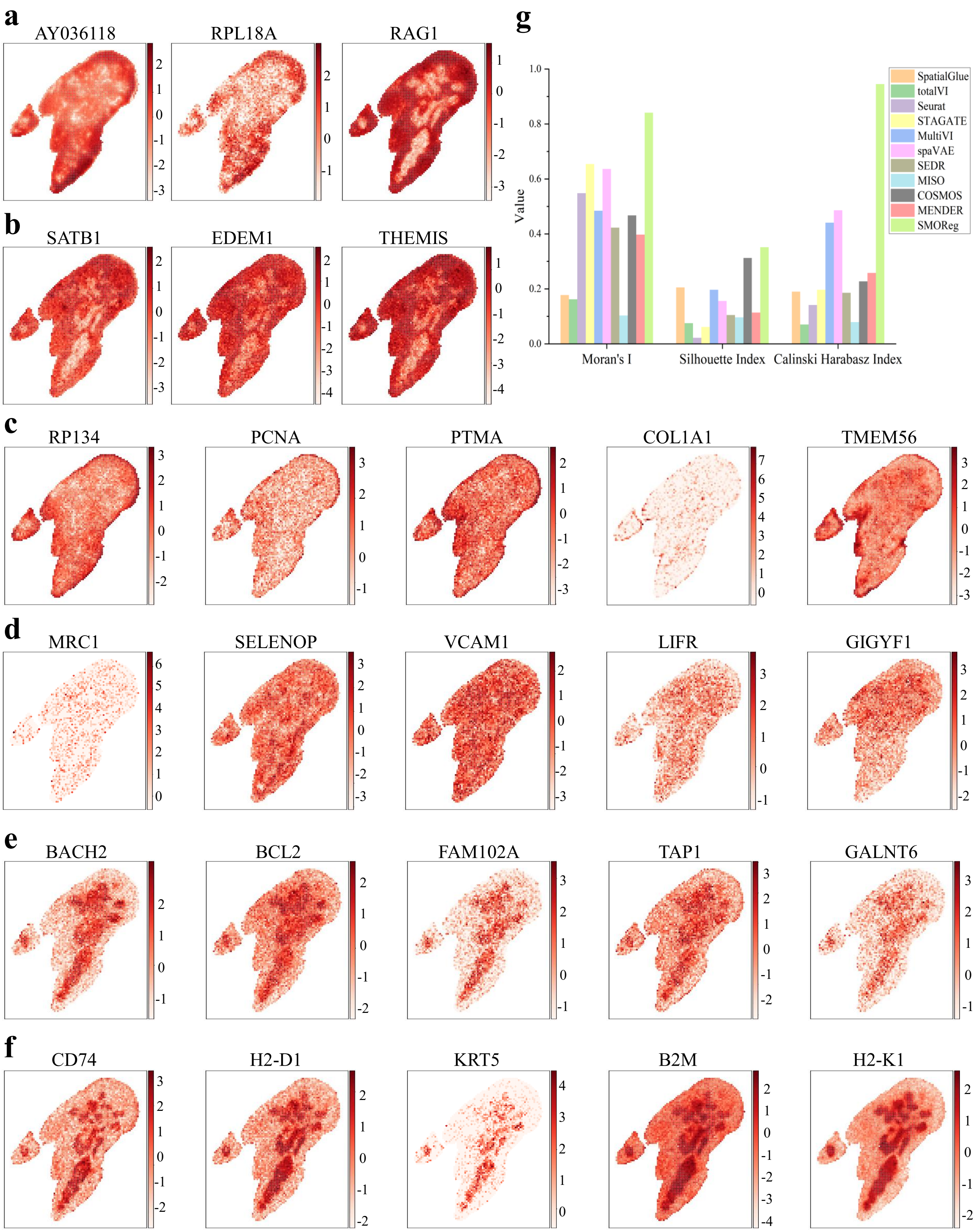
a
AY036118
RPL18A
RAG1
b
SATB1
EDEM1
THEMIS
c
RP134
PCNA
PTMA
COL1A1
TMEM56
d
MRC1
SELENOP
VCAM1
LIFR
GIGYF1
e
BACH2
BCL2
FAM102A
TAP1
GALNT6
f
CD74
H2-D1
KRT5
B2M
H2-K1
g
Value
1.0
0.8
0.6
0.4
0.2
0.0
Moran's I
Silhouette Index
Calinski Harabasz Index
SpatialGlue
totalVI
Seurat
STAGATE
MultiVI
spaVAE
SEDR
MISO
COSMOS
MENDER
SMOReg

Supplementary Fig. 12: Spatial expression heatmaps of representative genes differentially expressed in each spatial domain of the mouse thymus sample. **a**, SMOReg cluster 1 (the outer cortex). **b**, SMOReg cluster 2 (the inter cortex). **c**, SMOReg cluster 3 (the capsular zone). **d**, SMOReg cluster 4 (the thymic nurse cell zone). **e**, SMOReg cluster 5 (the cortico-medullary junction). **f**, SMOReg cluster 6 (the medulla region). The color intensity reflects the relative expression level of each molecule, quantified as z-scores after data scaling, with darker shades indicating higher standardized values. **g**, Bar plot comparing clustering evaluation scores of SMOReg and ten baselines across three metrics (mouse thymus sample): Moran's I, Silhouette Index, and Calinski-Harabasz Index. Higher values indicate better performance.

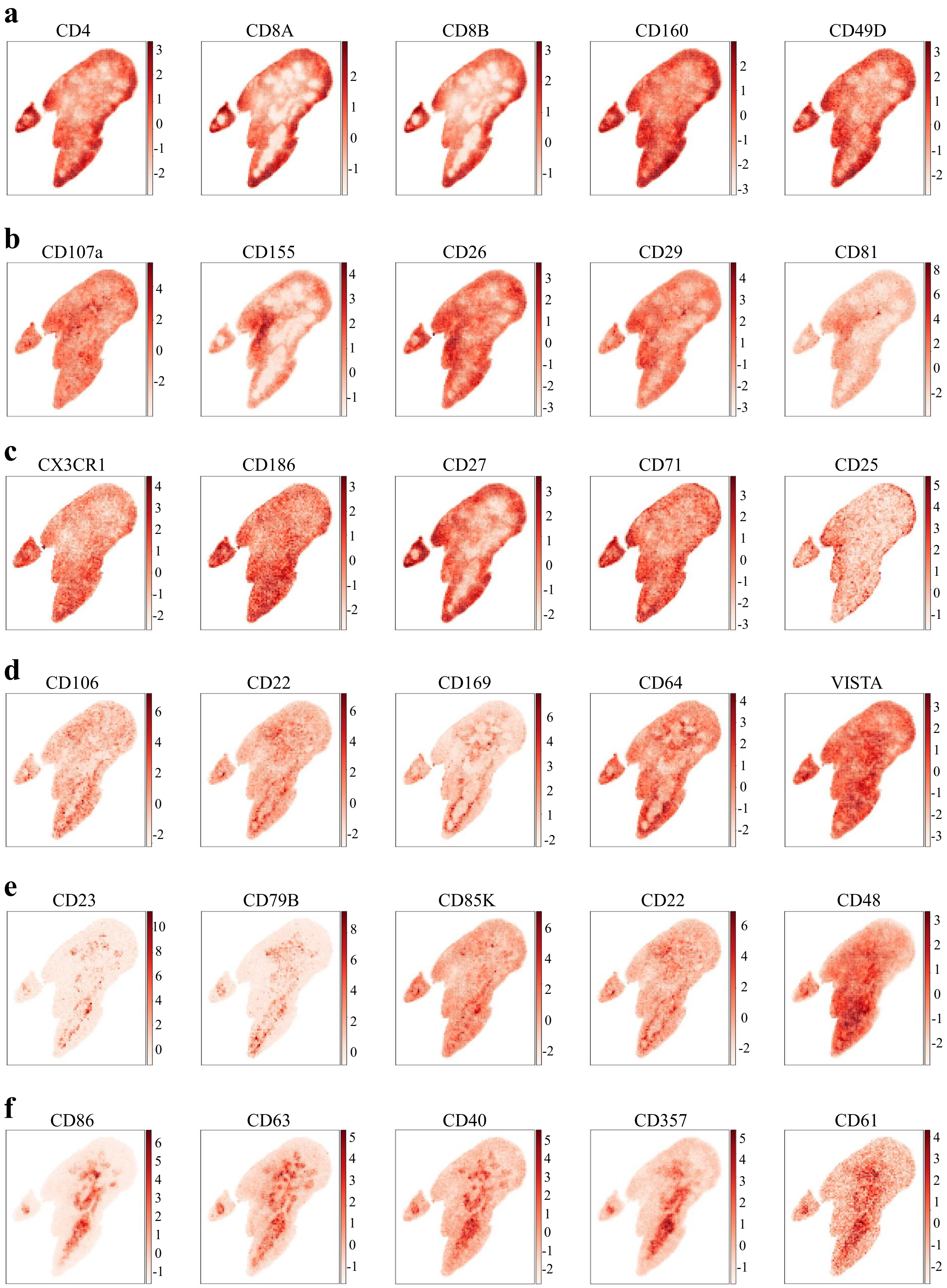
a
CD4
CD8A
CD8B
CD160
CD49D
b
CD107a
CD155
CD26
CD29
CD81
c
CX3CR1
CD186
CD27
CD71
CD25
d
CD106
CD22
CD169
CD64
VISTA
e
CD23
CD79B
CD85K
CD22
CD48
f
CD86
CD63
CD40
CD357
CD61

Supplementary Fig. 13: Spatial expression heatmaps of representative proteins differentially expressed in each spatial domain of the mouse thymus sample. **a**, SMOReg cluster 1 (the outer cortex). **b**, SMOReg cluster 2 (the inter cortex). **c**, SMOReg cluster 3 (the capsular zone). **d**, SMOReg cluster 4 (the thymic nurse cell zone). **e**, SMOReg cluster 5 (the cortico-medullary junction). **f**, SMOReg cluster 6 (the medulla region). The color intensity reflects the relative abundance level of each molecule, quantified as z-scores after data scaling, with darker shades indicating higher standardized values.

a

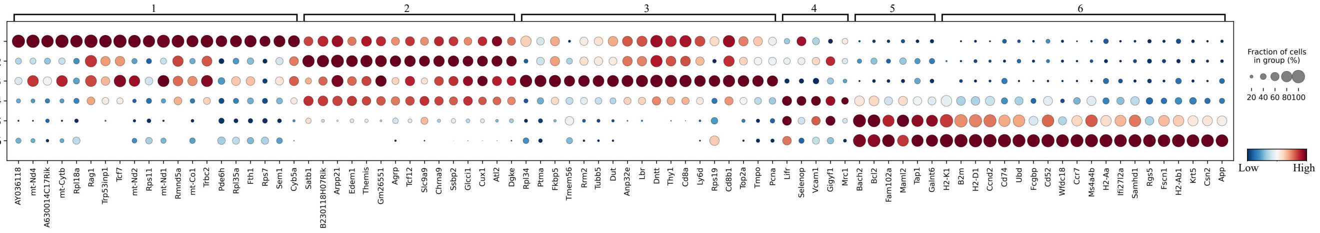

b

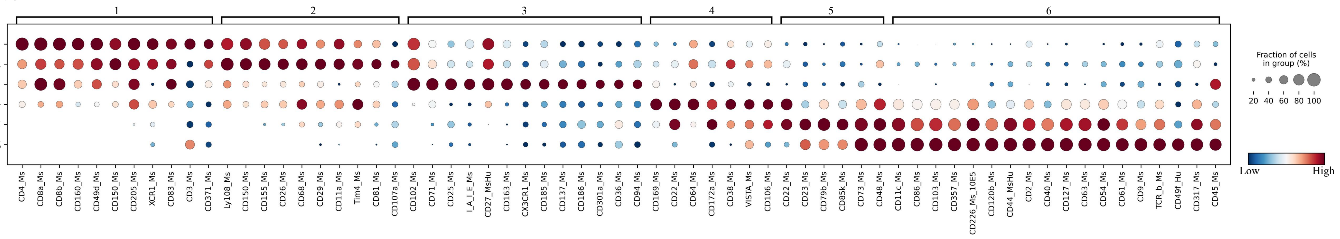

c

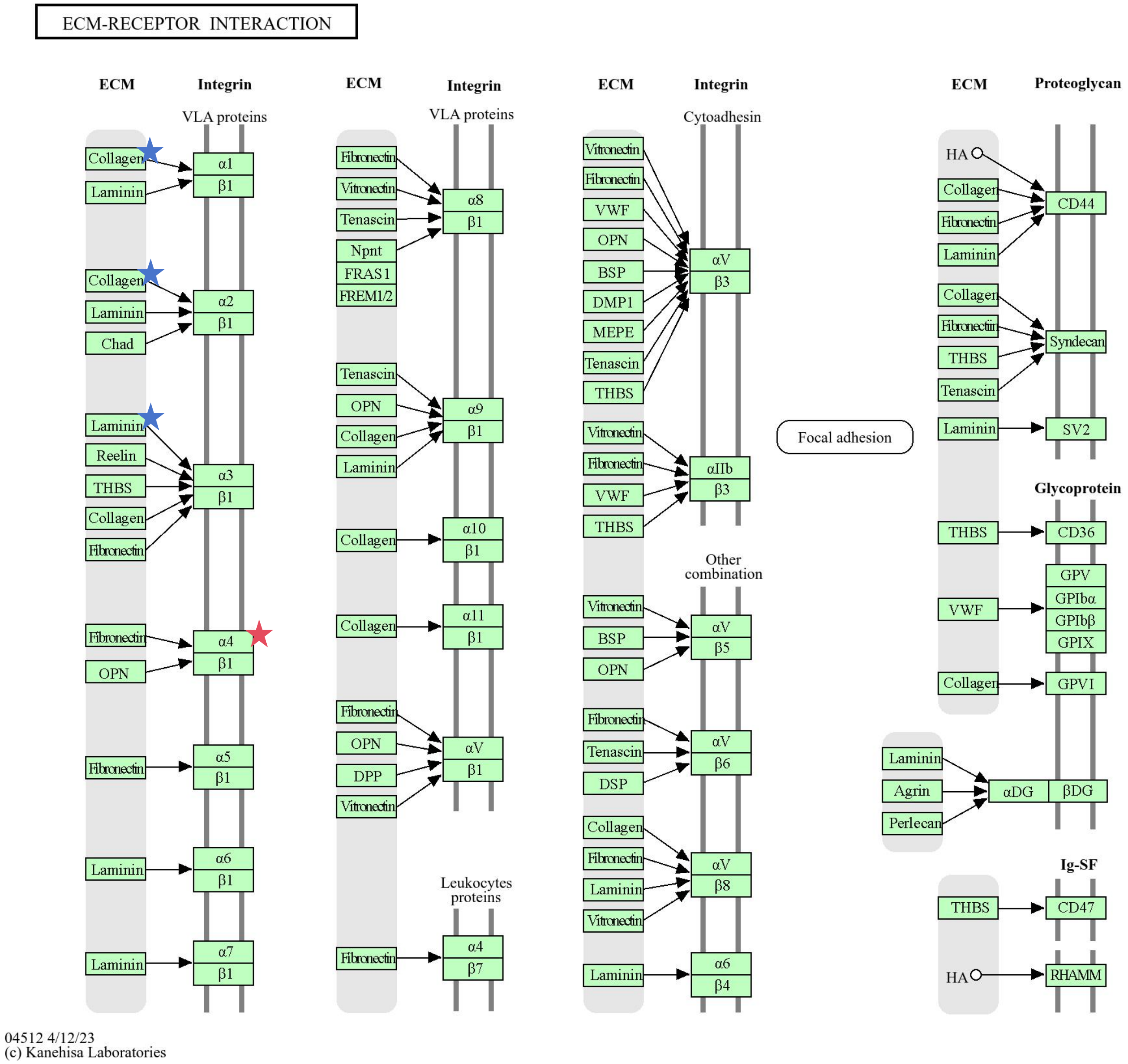
ECM-RECEPTOR INTERACTION
ECM
Integrin
VLA proteins
Collagen
Laminin
α1
β1
Collagen
Laminin
Chad
α2
β1
Laminin
Reelin
THBS
Collagen
Fibronectin
α3
β1
Fibronectin
OPN
α4
β1
Fibronectin
α5
β1
Laminin
α6
β1
Laminin
α7
β1
ECM
Integrin
VLA proteins
Fibronectin
Vitronectin
Tenascin
Npnt
FRAS1
FREM1/2
α8
β1
Tenascin
OPN
Collagen
Laminin
α9
β1
Collagen
α10
β1
Collagen
α11
β1
Fibronectin
OPN
DPP
Vitronectin
αV
β1
Leukocytes proteins
Fibronectin
α4
β7
ECM
Integrin
Cytoadhesin
Vitronectin
Fibronectin
VWF
OPN
BSP
DMP1
MEPE
Tenascin
THBS
αV
β3
Vitronectin
Fibronectin
VWF
THBS
αIIb
β3
Other combination
Vitronectin
BSP
OPN
αV
β5
Fibronectin
Tenascin
DSP
αV
β6
Collagen
Fibronectin
Laminin
Vitronectin
αV
β8
Laminin
α6
β4
Focal adhesion
ECM
Proteoglycan
HA
Collagen
Fibronectin
Laminin
CD44
Collagen
Fibronectin
THBS
Tenascin
Syndecan
Laminin
SV2
Glycoprotein
THBS
CD36
VWF
GPV
GPIbα
GPIbβ
GPIX
Collagen
GPVI
Laminin
Agrin
Perlecan
αDG
βDG
Ig-SF
THBS
CD47
HA
RHAMM
04512 4/12/23
(c) Kanehisa Laboratories

Supplementary Fig. 14: Additional results for mouse thymus analysis. **a**, Transcriptomic differential expression for SMOReg clusters. Dot size represents expressing cell fraction; color indicates the average expression level. **b**, Proteomic differential expression across the same clusters. **c**, KEGG pathway map (hsa04512) of the ECM–receptor interaction pathway. This diagram illustrates the interactions between extracellular matrix (ECM) components and integrin receptors. Blue stars indicate proteins from the collagen family that serve as ECM ligands, while the red star marks the integrin subunit *ITGA4*.

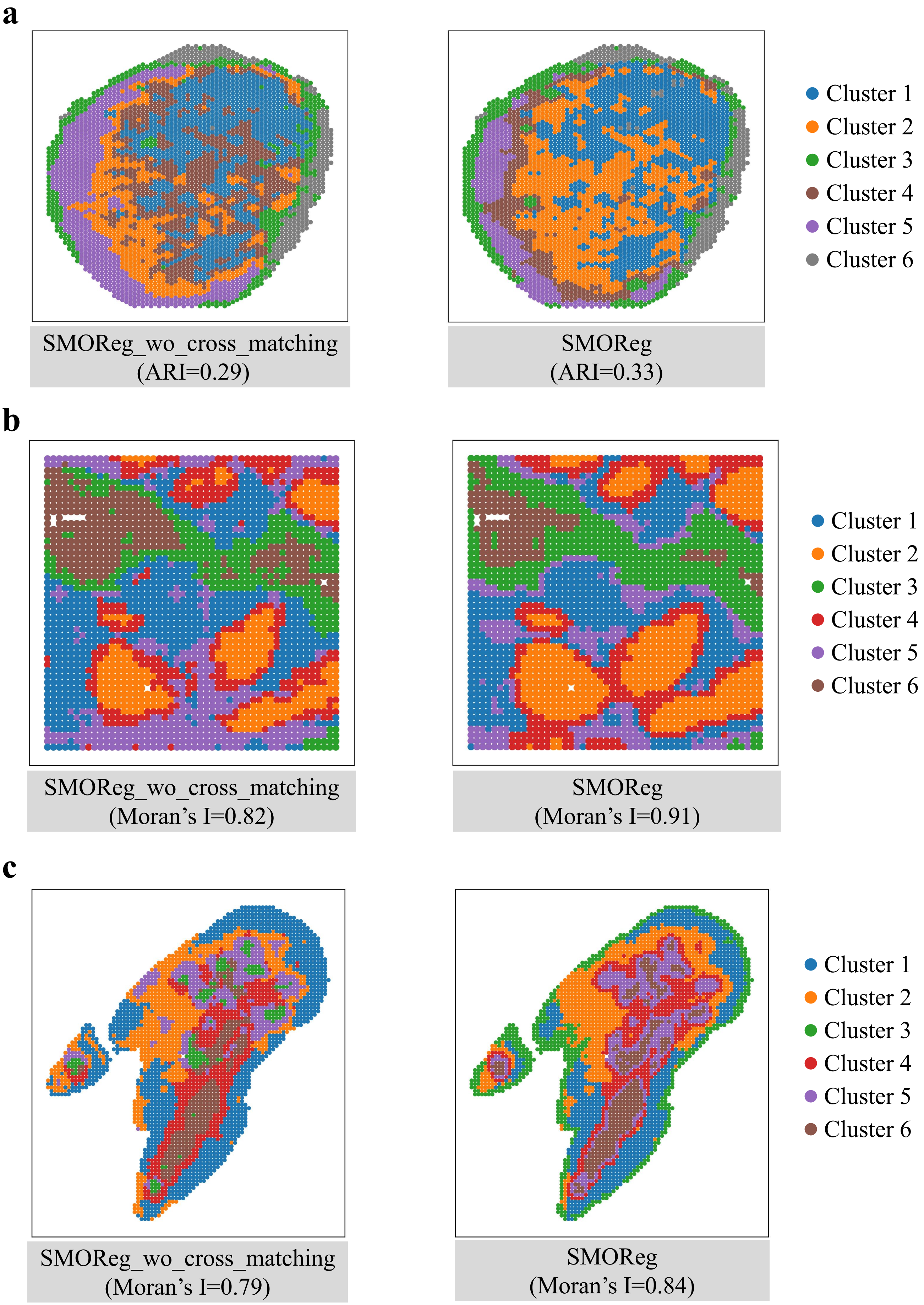
a
Cluster 1
Cluster 2
Cluster 3
Cluster 4
Cluster 5
Cluster 6
SMOReg_wo_cross_matching
(ARI=0.29)
SMOReg
(ARI=0.33)
b
Cluster 1
Cluster 2
Cluster 3
Cluster 4
Cluster 5
Cluster 6
SMOReg_wo_cross_matching
(Moran’s I=0.82)
SMOReg
(Moran’s I=0.91)
c
Cluster 1
Cluster 2
Cluster 3
Cluster 4
Cluster 5
Cluster 6
SMOReg_wo_cross_matching
(Moran’s I=0.79)
SMOReg
(Moran’s I=0.84)

Supplementary Fig. 15: Qualitative ablation study comparing spatial domain identification with and without the cross-graph matching module. Spatial clustering results of the baseline model (SMOReg_wo_cross_matching) versus the full model (SMOReg) across three real tissue datasets. **a**, Human lymph node dataset. The ablated model fails to separate the cortex and paracortex, incorrectly over-clustering the central medulla. **b**, Human tonsil dataset. Without cross-graph matching, the boundaries between the germinal center zones and surrounding tissues become blurred, introducing scattered noise points. **c**, Mouse thymus dataset. The ablated model struggles to maintain the correct hierarchical structure, losing the outermost capsular layer and mixing the medullary regions. Across all tissues, the full SMOReg model yields significantly sharper boundaries, higher spatial coherence, and better preservation of subtle anatomical microenvironments.

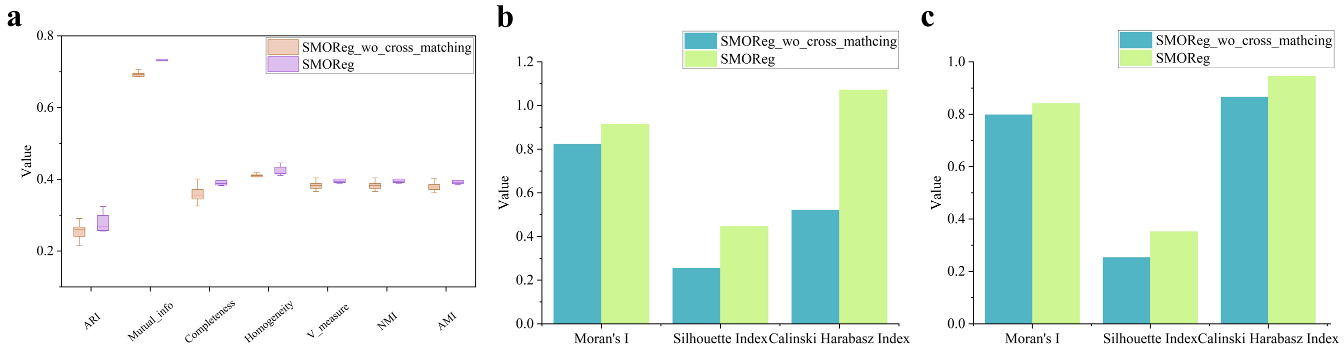

Supplementary Fig. 16: Quantitative ablation study assessing the contribution of the cross-graph matching module to clustering metrics. Performance comparison between the ablated baseline (SMOReg_wo_cross_matching) and the full framework (SMOReg). **a**, Boxplots of seven supervised clustering metrics (e.g., ARI, Mutual Information, Completeness) on the annotated human lymph node dataset. The full model demonstrates not only significantly higher median scores but also narrower interquartile ranges, indicating superior accuracy and stability. **b**, Bar charts of three unsupervised metrics (Moran's I, Silhouette Index, Calinski-Harabasz Index) on the human tonsil dataset. **c**, Bar charts of unsupervised metrics on the mouse thymus dataset. In both unsupervised evaluations (**b** and **c**), the integration of the cross-graph matching module provides substantial quantitative gains, verifying its essential role in enhancing overall model performance.

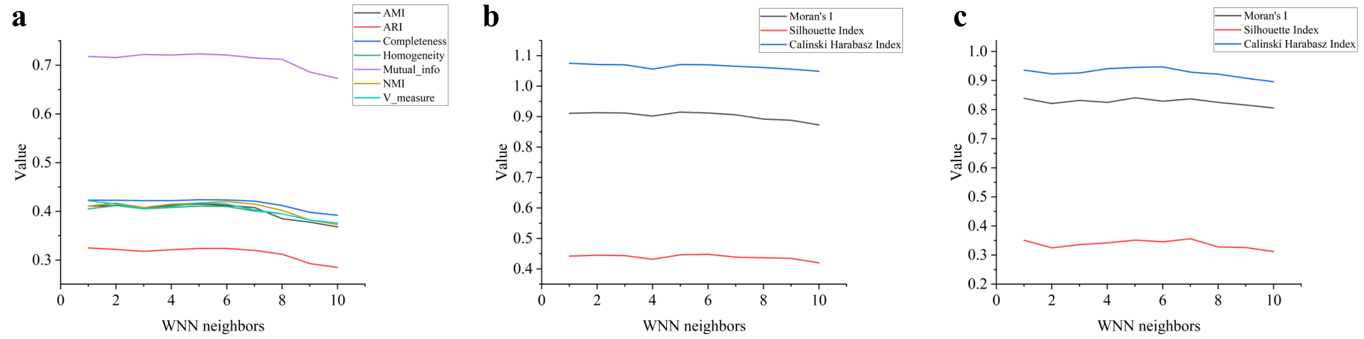

Supplementary Fig. 17: Sensitivity analysis of spatial domain identification performance to the number of WNN neighbors. The line charts display the evaluation metrics for spatial domain clustering on real datasets as the number of neighbors (k) in the Weighted Nearest Neighbor (WNN) analysis varies from 1 to 10. **a–c**, Performance trends across different real tissue samples, specifically the human lymph node (**a**), human tonsil (**b**), and mouse thymus (**c**). The metrics (e.g., ARI, AMI, Moran's I, etc.) remain largely stable for $k \leq 7$, peaking optimally around $k = 5$, demonstrating the robustness of SMOReg to graph-building hyperparameters. The slight decline at higher values ($k \geq 8$) reflects the expected inclusion of less biologically similar neighbors inherent to the k-NN search logic.

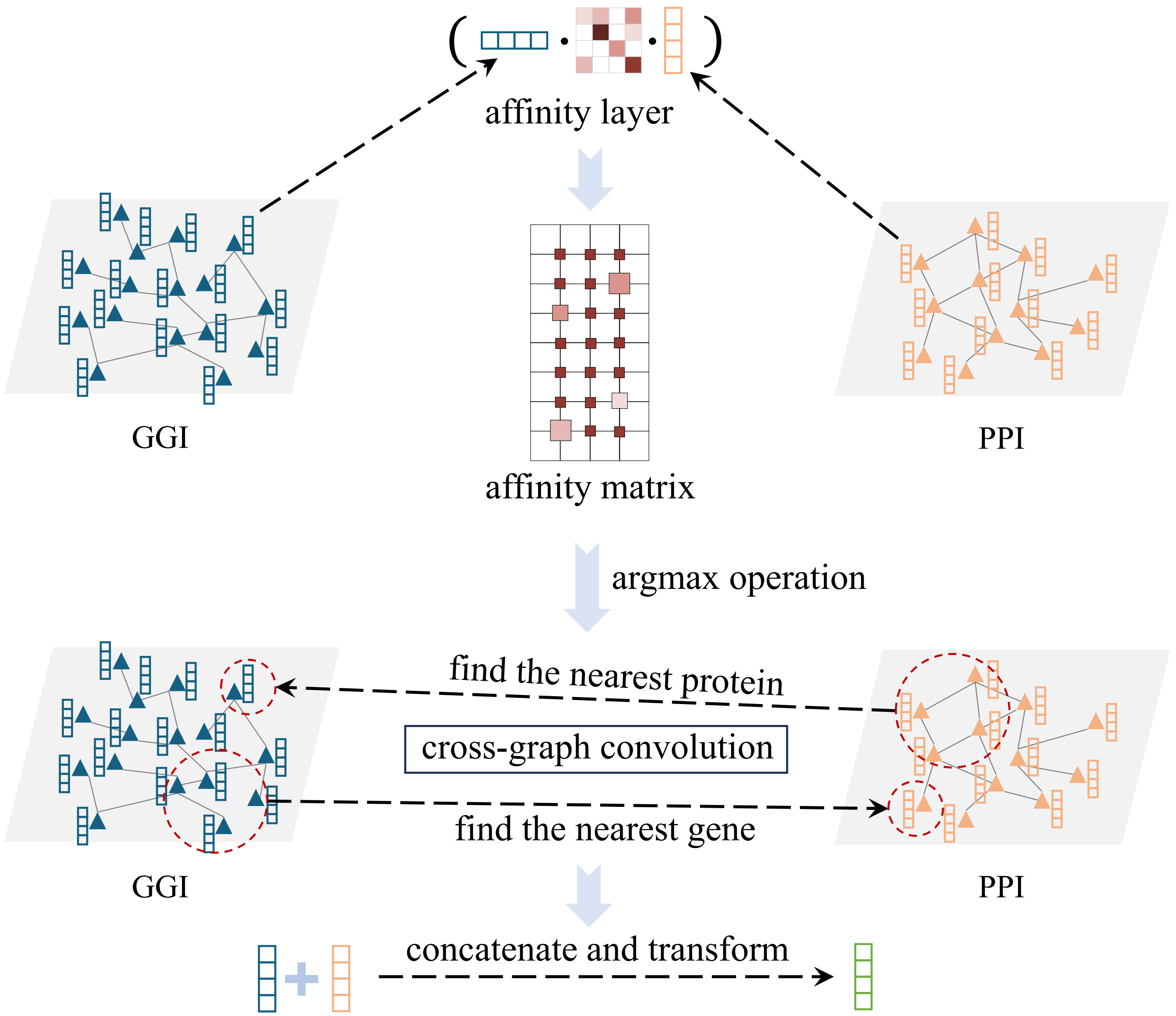

Supplementary Fig. 18: Detailed architectural schematic of the cross-graph matching mechanism. This data flow diagram illustrates the specific algorithmic operations corresponding to Equations 3–5 in the SMOReg framework. **Top**: The intra-omics representations from the Gene-Gene Interaction (GGI) and Protein-Protein Interaction (PPI) graphs are processed through an affinity layer to compute a pairwise affinity matrix, estimating cross-omics interaction strengths. **Middle**: During the cross-graph convolution step, an argmax operation is applied to the affinity matrix to identify and align the most relevant cross-omics neighbors (e.g., mapping a specific gene to its nearest protein, and vice versa, highlighted by red dashed circles). **Bottom**: The features of these selected high-affinity nodes are then extracted, concatenated with the original node representations, and transformed via a linear update function to generate refined, multi-omics-aware molecular embeddings.
